# Associations of child and adolescent anxiety with later alcohol use and disorders: a systematic review and meta‐analysis of prospective cohort studies

**DOI:** 10.1111/add.14575

**Published:** 2019-03-19

**Authors:** Maddy L. Dyer, Kayleigh E. Easey, Jon Heron, Matthew Hickman, Marcus R. Munafò

**Affiliations:** ^1^ UK Centre for Tobacco and Alcohol Studies, School of Psychological Science University of Bristol Bristol UK; ^2^ Population Health Sciences, Bristol Medical School University of Bristol Bristol UK; ^3^ MRC Integrative Epidemiology Unit (IEU) University of Bristol Bristol UK

**Keywords:** Adolescence, alcohol, anxiety, childhood, longitudinal, meta‐analysis, systematic review, young adulthood

## Abstract

**Background and Aims:**

Despite a wealth of literature, the relationship between anxiety and alcohol use remains unclear. We examined whether (a) child and adolescent anxiety is positively or negatively associated with later alcohol use and disorders and (b) study characteristics explain inconsistencies in findings.

**Design and Setting:**

We conducted a systematic review of 51 prospective cohort studies from 11 countries. Three studies contributed to a meta‐analysis. We searched PubMed, Scopus, Web of Science and PsycINFO databases, and studies were included if they met the following criteria: English language publication, human participants, anxiety exposure (predictor variable) in childhood or adolescence and alcohol outcome at least 6 months later.

**Participants:**

Study sample sizes ranged from 110 to 11 157 participants. Anxiety exposure ages ranged from 3 to 24 years, and alcohol outcome ages ranged from 11 to 42 years.

**Measurements:**

Ninety‐seven associations across 51 studies were categorized by anxiety exposure (generalized anxiety disorder, internalizing disorders, miscellaneous anxiety, obsessive compulsive disorder, panic disorder, separation anxiety disorder, social anxiety disorder and specific phobias) and alcohol use outcome (drinking frequency/quantity, binge drinking and alcohol use disorders).

**Findings:**

The narrative synthesis revealed some evidence for a positive association between anxiety and later alcohol use disorders. Associations of anxiety with later drinking frequency/quantity and binge drinking were inconsistent. Type and developmental period of anxiety, follow‐up duration, sample size and confounders considered did not appear to explain the discrepant findings. The meta‐analysis also showed no clear evidence of a relationship between generalized anxiety disorder and later alcohol use disorder (odds ratio = 0.94, 95% confidence interval = 0.47–1.87).

**Conclusions:**

Evidence to date is suggestive, but far from conclusive of a positive association between anxiety during childhood and adolescence and subsequent alcohol use disorder.

## Introduction

There is considerable clinical and research interest in determining the nature of associations between anxiety and alcohol disorders, including their strength and direction, given the substantial health, social and economic costs associated with both conditions 1, 2, 3. However, despite a wealth of observational evidence, the relationship between anxiety and alcohol use remains unclear.

Different theories exist regarding the temporal sequence and directionality of the relationship, and evidence is inconsistent 4. First, the self‐medication hypothesis suggests that anxious individuals consume alcohol to alleviate their physiological and emotional reactivity 5, 6. Secondly, anxiety may be protective due to social withdrawal, fear of negative consequences associated with drinking 7 and concerns about cognitive or behavioural impairment 8, 9. Thirdly, chronic alcohol use may cause anxiety, via biological or psychosocial mechanisms 10. Finally, there may be no causal relationship between anxiety and alcohol use; any associations found may be a product of shared risk factors or confounding.

There are several possible explanations as to why the literature is conflicting. First, anxiety is heterogeneous; different anxiety disorders or symptoms may be associated with unique patterns of drinking. For example, Fröjd and colleagues 11 found general anxiety was associated with a higher incidence of frequent alcohol use; however, social phobia was associated with a lower incidence. Furthermore, Nichter & Chassin 12 found that adolescent physiological anxiety increased the risk of binge drinking and alcohol dependence, whereas worry was associated with a decreased risk. Secondly, variability in alcohol‐related phenotypes may explain inconsistent findings. For example, adolescent social anxiety disorder and panic disorder predicted alcohol dependence in early adulthood, but not alcohol abuse 13. It is therefore important to consider how authors operationalize anxiety and alcohol use. Thirdly, the relationship may be age‐dependent. For instance, there is some evidence that child internalizing symptoms are negatively associated with adolescent alcohol use 14, whereas adolescent anxiety is positively associated with adult alcohol use adulthood 15. Some researchers suggest that the self‐medication pathway may only develop in late adolescence/early adulthood 16, which may explain these differences. Fourthly, authors may not have adequately adjusted for confounders, or other sources of bias may have caused spurious findings. Finally, other variables could influence the strength and direction of the anxiety–alcohol relationship; anxiety may act as a risk or protective factor if there are moderating influences 17.

Although there have been numerous critical reviews on this topic 4, 18, 19, 20, 21, 22, 23, only a few systematic reviews and meta‐analyses have been conducted. In one meta‐analysis, social anxiety among college students was negatively associated with alcohol use but positively associated with alcohol‐related problems 9. This reinforces the importance of examining the relationship separately for different alcohol outcomes, although direction of effect could not be inferred, as this was a review of cross‐sectional studies. Groenman and colleagues 24 found that childhood anxiety disorders did not increase the risk for later alcohol disorders. However, the authors acknowledged that findings from individual studies were highly heterogeneous, and only five studies on anxiety and alcohol use were included. In a review of longitudinal studies which adjusted for co‐occurring externalizing symptoms, Hussong and colleagues 25 also found no clear association between anxiety and internalizing symptoms with subsequent adolescent alcohol use. A limitation of this review was that authors counted some non‐independent associations, which may have distorted the results. It is important to consider whether studies account for confounders, including other psychiatric problems (e.g. externalizing disorders), and other factors such as gender, as these may be a source of bias. For example, there is evidence that externalizing disorders and being female are positively associated with anxiety 26, 27, and externalizing disorders and being male are positively associated with alcohol use 28, 29. Therefore, if externalizing disorders and gender are not statistically adjusted for, they may cause spurious associations between anxiety and alcohol use.

In the current systematic review, we synthesized evidence from cohort studies investigating prospective associations between child and adolescent anxiety with later alcohol use outcomes. We examined whether (a) anxiety is positively or negatively associated with later alcohol use and (b) study characteristics explain any inconsistences in findings (i.e. type and developmental period of anxiety, type of alcohol use, length of follow‐up, sample size and confounders adjusted for). We restricted the review to prospective studies to improve inferences about the chronology of anxiety and alcohol use. While important, associations between alcohol use and subsequent anxiety were not examined for practical and theoretical reasons. We were primarily interested in whether anxiety disorders were a risk factor for later alcohol use and disorders, in line with the self‐medication hypothesis. We also performed a meta‐analysis on a small subgroup of comparable studies. By detecting patterns across multiple study characteristics, we aimed to identify which individuals may be more at risk of greater alcohol use and disorders. Currently, the discrepant evidence prevents the development of tailored prevention and intervention programmes.

## Methods

This review was pre‐registered on the Open Science Framework (https://osf.io/vg39k/) and all applicable PRISMA (Preferred Reporting Items for Systematic Reviews and Meta‐Analyses) and MOOSE (Meta‐analyses Of Observational Studies in Epidemiology) guidelines were followed.

### Selection criteria

Studies were included if they met the following criteria: English language peer‐reviewed publication, human participants, anxiety exposure in childhood (< 10 years) or adolescence (≥ 10 and < 18 years), alcohol outcome(s) distinct from general substance use and measured at least 6 months later than exposure and longitudinal design. Anxiety exposure refers to any anxiety measure used as a predictor variable (i.e. it preceded the alcohol use outcome by at least 6 months). If an anxiety exposure range extended beyond age 18 years but included adolescence (e.g. 14–24 years), we still included the study. However, if the study sample range was solely or predominantly above 18 years, we excluded the study. We did not have the resources to translate non‐English language publications and locate unpublished studies. ‘Studies’ refer to published journal articles.

### Identification of studies

We searched PubMed, Scopus, Web of Science and PsycINFO electronic databases until February 2017, using the following terms: anxi*, internali?ing, phobi*, *phobia, panic, obsessive–compulsive, OCD, post‐traumatic stress disorder, PTSD, alcohol*, drink*, ethanol, longitudinal, prospective, cohort, trajector*, wave (see Supporting information for an example). One author (M.D.) first screened electronic titles, abstracts and keywords, then full‐text articles. Reasons for exclusion at the second phase were documented. A 10% check was independently completed by a second author (K.E.) at each screening phase. Any disagreements were resolved by consensus. M.D. also hand‐searched reference lists of included articles.

We later excluded post‐traumatic stress disorder, because it has been reclassified in the Diagnostic and Statistical Manual of Mental Disorders (5th edition). Studies were excluded if alcohol initiation was the only outcome, as we were primarily interested in level, rather than commencement of use. Finally, studies were excluded if statistical analyses violated our inclusion criteria (e.g. concurrent or retrospective analyses). See Supporting information, Table S1 for excluded studies.

### Data extraction

M.D. extracted the following information from each included study: sample, country, percentage male, anxiety exposure (measure, age, respondent), alcohol use outcome (measure, age, respondent), follow‐up time, statistical test, results, confounders adjusted for and sample size. Full data extraction was independently checked by a second author (K.E.) to help minimize errors. Differences were resolved by consensus.

### Quality assessment

We assessed methodological quality by focusing on whether authors adjusted for important potential confounders. All studies had an appropriate follow‐up period, as we pre‐specified this. We did not perform a formal risk of bias assessment, as this is typically used to explore heterogeneity in a meta‐analysis.

### Classification and synthesis of research findings

As anticipated, there was considerable heterogeneity between studies in terms of type and age of anxiety exposure and alcohol outcome, length of follow‐up, statistical methods and confounders adjusted for. This diversity precluded a statistical synthesis of findings from all 51 studies. We therefore present a narrative summary of results.

We coded associations according to strength of evidence: negative [negative point estimate and *P* < 0.05 or 95% confidence interval (CI) excludes the null], weak negative (negative point estimate and *P* < 0.1 or > 70% of the 95% CI is in the negative direction), equivocal (*P* > 0.1 or < 70% of the 95% CI is in a positive or negative direction), weak positive (positive point estimate and *P* < 0.1 or > 70% of the 95% CI is in a positive direction, positive (positive point estimate and *P* < 0.05 or 95% CI excludes null) and unclassifiable (required statistical information not reported).

Anxiety exposures and alcohol outcomes were grouped based on behavioural and clinical similarity. We organized associations based on three alcohol use categories: drinking frequency/quantity (hazardous drinking, heavy drinking, drinking frequency, alcohol quantity and alcohol use), binge drinking (binge drinking, heavy episodic drinking and intoxication/drunkenness) and alcohol use disorders (AUD; alcohol dependence, alcoholism, harmful drinking, alcohol use disorders, alcohol problems and alcohol abuse). We subcategorized by eight anxiety categories: generalized anxiety disorder (GAD) (including overanxious disorder and general worry), internalizing disorders (including anxiety/depression combined), obsessive compulsive disorder (OCD), panic disorder (including panic attacks), separation anxiety disorder, social anxiety disorder (including social phobia) and specific phobias. We also had a miscellaneous anxiety category that included trait measures (behavioural inhibition, trait anxiety and anxiety sensitivity) and combined measures of several anxiety disorders. We counted the number of positive, negative, equivocal and unclassifiable associations according to type of anxiety exposure and alcohol outcome.

As many studies reported several associations, we devised rules to avoid counting non‐independent associations. For each study, and for each anxiety exposure, only one drinking frequency/quantity, one binge drinking and one AUD outcome association were counted. If several alcohol outcomes were reported from the same alcohol category, we selected based on the order they are listed above (e.g. alcohol dependence instead of AUD). Other decision rules were: most adjusted result, unstandardized betas (versus standardized), main effects (versus interactions), male and female results if total not reported, adolescent anxiety (versus child), alcohol use in early adulthood (versus other developmental periods), more complex model (versus simpler model), adolescent report (versus parent versus teacher), parent report (versus teacher versus child), panic attacks (versus panic disorder), anxiety prior year (versus baseline), anxiety > 2 waves (versus 1–2 waves), total anxiety score (versus subscales), greatest length of follow‐up (if several relevant time‐points reported), greatest class comparison (e.g. heavy use versus abstainers) and trajectories closest to our research question.

Finally, we performed a meta‐analysis on three studies investigating associations between GAD and AUD/alcohol dependence, due to similarity of exposure, outcome and statistical method. Studies were not included if they measured only worry, a different anxiety disorder, drinking frequency/quantity or binge drinking. One study that met our criteria was dropped, as the corresponding author did not respond to our request for statistical information. Results from the other seven anxiety categories were judged to be too heterogeneous for a meta‐analysis. M.D. judged the suitability of results for inclusion in the meta‐analysis after discussion with co‐authors. Statistical analyses were conducted in Stata version 15 using the *metan* command 30. Between‐study heterogeneity was assessed using *I* ^2^. As there was negligible heterogeneity, we used the DerSimonian and Laird method for fitting the random effects meta‐analysis model.

## Results

### Results of literature search

**Figure 1:**
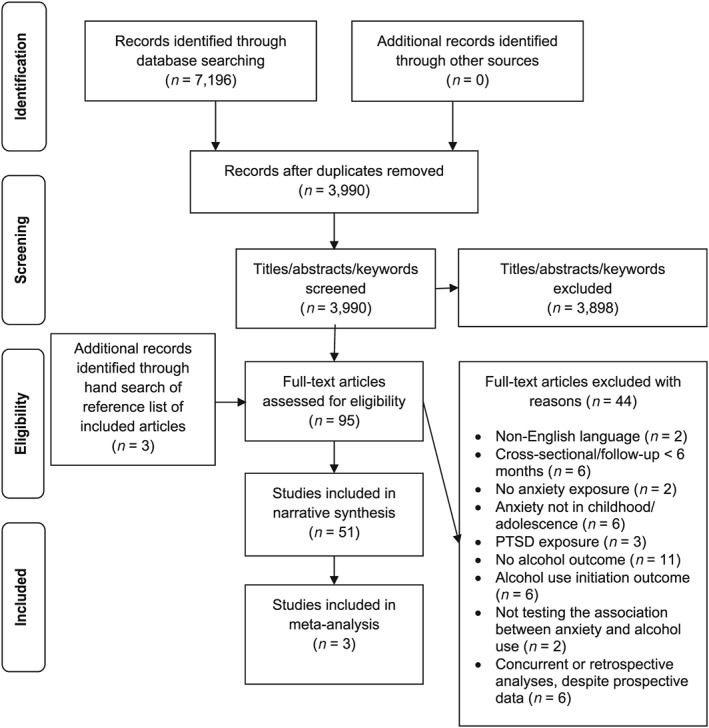
Preferred reporting items for systematic reviews and meta‐analyses (PRISMA) flow diagram of the literature search

A total of 3990 articles were screened by title/abstract/keywords. Ninety‐two full‐text articles were assessed, 44 of which were excluded. Three further articles were identified following a hand search of reference lists of the 48 included articles, leaving a total of 51 studies. Four associations from three studies contributed to the meta‐analysis (see Fig. 1).

### Characteristics of included studies

There were 27 studies from the United States, five from Germany and Finland, four from the United Kingdom, three from the Netherlands, two from Australia and one each from Taiwan, Canada, New Zealand, Sweden and Norway. Forty‐six studies included males and females, four had an all‐male sample and one had an all‐female sample. Thirty measures assessed anxiety and 40 measures assessed alcohol use. Length of follow‐up ranged from 6 months to 26 years, and sample sizes ranged from 110 to 11 157 participants. Age of anxiety and alcohol use ranged from 3 to 24 years and 11 to 42 years, respectively. See Supporting information, Table S2 for full data extraction information and Table 1 for a pared‐down version.

**Table 1:** Characteristics of included studies.

Study	Sample and country	Sample size	% Male	Anxiety type (measure)	Age of anxiety (years)	Alcohol use type (measure)	Age of alcohol use (years)
1. 31	Youth at a juvenile detention centre, USA	1504	64	Generalized anxiety disorder (DISC‐2.3)	10–19 (median 15)	Alcohol use disorder (DISC‐IV)	15–25 (median 20)
2. 32	Early Developmental Stages of Psychopathology Study, Germany	122	33	Panic attacks (DSM‐IV‐TR M‐CIDI)	14–24 (median 19)	Alcohol use disorder (DIA‐X/M‐CIDI)	21–34
3. 33	Early Developmental Stages of Psychopathology Study, Germany	2929	51	Specific phobias (DIA‐X/M‐CIDI)	14–24	Alcohol use, alcohol abuse, alcohol dependence (DIA‐X/M‐CIDI)	1.6, 3.5, and 8.2 years later
4. 34	Early Developmental Stages of Psychopathology Study, Germany	1090	49	Separation anxiety disorder: subthreshold, threshold (M‐CIDI)	14–17	Alcohol abuse, alcohol dependence (M‐CIDI)	20 and 42 months later
5. 13	Oregon Adolescent Depression Project, USA	816	41	Social, generalized and separation anxiety, panic, obsessive–compulsive and overanxious disorder, specific phobia (K‐SADS, K‐SADS‐P)	16	Alcohol abuse, alcohol dependence (LIFE, SCID‐4)	30
6. 35	Pittsburgh Youth Study, USA	487	100	Anxiety problems (CBCL, TRF, YSR, YASR)	13–19 (annual or semi‐annual)	Alcohol frequency, alcohol quantity (Substance Use Scale from NYS)	13–19 (semi‐annual)
7. 36	Taiwan Aboriginal Study Project, Taiwan	164	30	Anxiety disorders (Chinese version of the CIS)	15–24	Time to onset of alcoholism (Chinese version of the CIS)	4 years later
8. 37	From a longitudinal study of adolescent substance use, USA	367	45	Internalizing problems (YSR)	11–13	Alcohol use (NYS)	12–16
9. 38	From a longitudinal study of HIV‐related risk behaviours, USA	277	56	Social phobia (RCADS)	11, 12, 13, 14, 15	Alcohol use (modified version of YRBSS)	11, 12, 13, 14, 15
10. 14	Avon Longitudinal Study of Parents and Children, UK	11 157	51	Internalizing symptom trajectories (SDQ)	3, 6, 8, 9, 11	Whole drink, drank without parental permission, ever binge, number of whole drinks in past 6 months	13
11. 39	Minnesota Longitudinal Study of Parents and Children, USA	158–170	53	Internalizing behaviour (TRF of Child Behavior Checklist)	9	Abstainers, moderate drinkers, heavy drinkers, and alcohol use disorder (Adult Health Survey)	19, 23, 26, 28
12. 40	Minnesota Longitudinal Study of Parents and Children, USA	191	55	Internalizing symptoms (TRF, YSR)	7, 9, 12, 16	Frequency and quantity of alcohol use (Adolescent Health Survey)	16
13. 41	Oregon Adolescent Depression Project, USA	816	41	Anxiety disorders (K‐SADS, LIFE, SCID)	16, 17, 24, 30	Alcohol use disorder (K‐SADS, LIFE, SCID)	24, 30
14. 28	Oregon Adolescent Depression Project, USA	641	No info	Anxiety disorders (K‐SADS, LIFE, SCID‐NP)	16, 17, 24, 30	Alcohol use disorder (DSM III‐R, DSM‐IV, K‐SADS, LIFE, SCID‐NP)	16, 17, 24, 30
15. 42	Adolescent Mental Health Cohort Study, Finland	2070	44	General anxiety (1 item), social phobia (SPIN)	15–16	Frequent alcohol use, frequent drunkenness	17–18
16. 43	The British Child and Adolescent Mental Health Surveys, UK	3607	52	Internalizing symptoms (SDQ, DAWBA), internalizing disorder (clinical diagnosis)	11–12, 13–14, 15–16	Frequent alcohol consumption (different item for each group)	3 years later
17. 15	Early Developmental Stages of Psychopathology Study, Germany	2548	No info	Panic attacks (M‐CIDI)	14–24	Alcohol use disorder (M‐CIDI)	14–25 and 34–50 months later
18. 17	Oregon Adolescent Depression Project, USA	817	No info	Anxiety disorders (K‐SADS)	16	Alcohol use disorder (K‐SADS, LIFE)	16, 17, 24, 30
19. 44	From a longitudinal study of familial alcoholism, USA	166	62	Internalizing symptoms (CBCL, CDIS‐III‐R)	11–15	Alcohol problems (from Sher's 1987 questionnaire)	25
20. 45	Seattle Social Development Project, USA	640	50	Behavioural inhibition/trait anxiety (CBCL)	14–15	Alcohol abuse and alcohol dependence (DISC)	27
21. 46	Michigan Longitudinal Study, USA	1064	69	Distress/internalizing symptoms (YSR of CBCL)	12–14	Maximum number of drinks, heavy episodic drinking frequency (Drinking and Drug History questionnaire)	18–20
22. 47	Project on Human Development in Chicago Neighbourhoods, USA	724	51	Internalizing symptoms (YSR of CBCL)	12, 15, 18	Alcohol use (number of days drunk alcohol in the past month)	12, 15, 18
23. 48	Minnesota Twin Family Study, USA	699	0	Separation anxiety disorder, overanxious disorder (DICA‐R)	10–12 (mean 11)	Regular use, ever drunk, heavy drinking (DICA‐R)	14
24. 49	From 24 secondary schools in London with personality risk for substance misuse, UK	393	No info	Anxiety (BSI)	13, 13.5, 14, 14.5	Alcohol use (quantity × frequency)	13, 13.5, 14, 14.5
25. 50	National Child Development Study, UK	4756–12 772	52	Internalizing behaviours (Health and Behaviour Checklists)	7, 11	Weekly quantity and harmful drinking (CAGE)	16, 23, 33
26. 51	Healthy Schools and Drugs prevention programme, the Netherlands	853–979	48	Anxiety sensitivity (SURPS)	12–13 and 8, 20, 32 months later	Alcohol use and binge drinking	12–13, and 8, 20, and 32 months later
27. 52	Healthy Schools and Drugs prevention programme, the Netherlands	648–758	48	Anxiety sensitivity (SURPS)	12–13	Life‐time prevalence of alcohol use	20 months later
28. 53	Pittsburgh Youth Study, USA	503	100	Generalized anxiety and social anxiety (CBCL, YSR, TRF)	6	First alcohol problem (DIS)	20
29. 54	Camden Youth Development Study, USA	134	50	Social and generalized anxiety symptoms (SCARED)	11	Frequency of drinking alcohol	Every 4 months for 16 months
30. 55	From secondary schools in the state of Victoria, Australia	1758	No info	Anxiety/depression symptoms (CIS)	14–17 (6 waves every 6 months)	Alcohol abuse or dependence (CIDI)	24
31. 56	Northern Finland Birth Cohort 1986 Study, Finland	6349	49	Internalizing problems (Rutter Scales)	8	Often drunk	15–16
32. 12	The pathways to desistance project, juvenile offenders, USA	818	100	Worry, physiological anxiety (RCMAS)	14–19	Typical quantity of drinking, frequency of binge drinking, dependence	6 months later
33. 7	Pittsburgh Youth Study, USA	506	100	Anxiety/withdrawal (YSR, TRF, CBCL)	13	Alcohol abuse and dependence (DIS)	20, 25
34. 57	California Families Project, USA	620	50	Internalizing symptoms (MASQ)	14, 16	Frequency of alcohol use	14, 16
35. 58	From secondary special education schools, the Netherlands	378	88	Anxiety sensitivity (SURPS)	13	Alcohol use (quantity × frequency) and problems (trajectories)	2 year follow up (6–8 months between waves)
36. 59	Jyväskylä Longitudinal Study of Personality and Social Development, Finland	290–347	53	Anxiety (1 item)	8, 14	Heavy use, frequency of drinking, binge drinking, problem drinking (LSQ and interview questions)	20, 27, 42
37. 60	Jyväskylä Longitudinal Study of Personality and Social Development, Finland	242–311	53	Anxiety (3 items)	8, 14	Social drinking, problem drinking, controlled drinking (CAGE)	26
38. 61	Finn Twin12 study, Finland	1225–1906	51	Social anxiety (MPNI)	12	Drinking frequency, alcohol dependence (SSAGA)	14, 17, 22
39. 62	Community sample, USA	387	45	Internalizing problems (YSR)	11–12	Alcohol use (YSR of Achenbach assessment)	12–13, 13–14
40. 63	From a primary prevention study, USA	295	39	Anxiety sensitivity (ASI), trait anxiety (STPI)	16–24	Alcohol use disorder (SCID‐NP)	18–26
41. 64	American Indian Research data, USA	281	No info	Internalizing behaviours (CBCL)	11	Alcohol use disorder (SSAGA‐II)	19–20
42. 65	Community sample, USA	185–187	47	Internalizing behaviour problems (RBPC)	11–15	Alcohol use (MAST, NYS)	17–22
43. 66	Longitudinal community sample (1/2 parental alcoholism), USA	216	52	Internalizing symptoms (CBCL)	12–16	Quantity and frequency of alcohol use, problem alcohol use	13–17
44. 67	Young‐HUNT 1, and Young‐HUNT 2, Norway	2399	46	Anxiety/depression (SCL 90‐R, SCL‐5)	13–15	Frequent alcohol use	17–19
45. 68	Random sample from secondary schools, Australia	1203	50	Anxiety/depression symptoms (CIS‐R)	14/15–17 (2 waves every 6 months)	Alcohol use disorder symptom classes (CIDI)	24
46. 69	Victoria Healthy Youth Survey, Canada	657–662	49	Internalizing symptoms (BCFPI)	12/13, 14/15, 16/17	Heavy episodic drinking, alcohol related harms (Harmful Effects of Alcohol Scale)	12/13, 14/15, 16/17, 18/19
47. 70	The Northern Swedish Cohort Study, Sweden	1010	52	Anxiousness (DSM‐5)	16	Drinking trajectories (frequency, consumption)	16, 18, 21, 30, 42
48. 71	Black adolescents with asthma, USA	110	34	Anxiety symptoms (MASC‐10)	11–19	Alcohol use frequency (from Adolescent Risk Behavior Survey)	12–20
49. 72	Northwestern‐UCLA Youth Emotion Project, USA	420–627	31	Anxiety disorders (SCID‐I/NP)	16	Alcohol use disorder (SCID‐I/NP)	1–4 years later
50. 73	Christchurch Health and Development Study, New Zealand	964	50	Anxiety disorders (DISC supplemented by DSM‐III‐R)	15–16	Alcohol abuse/dependence (CIDI)	Between 16 and 21, annually
51. 74	Early Developmental Stages of Psychopathology Study, Germany	2548	No info	Anxiety disorders (DIA‐X/M‐CIDI)	14–24	Regular use, hazardous use, abuse, dependence, alcohol use disorder (M‐CIDI)	20 and 42 months later

Anxiety measures: Diagnostic Interview Schedule for Children (DISC): 3; Munich‐Composite International Diagnostic Interview (M‐CIDI): 5; Kiddie Schedule for Affective Disorders and Schizophrenia (K‐SADS): 4; Achenbach System of Empirically Based Assessment (ASEBA), Child Behaviour Checklist (CBCL)/Youth Self‐Report (YSR)/Teacher's Report Form (TRF)/Young Adult Self‐Report (YASR): 13; Clinical Interview Schedule (CIS)/Clinical Interview Schedule‐Revised (CIS‐R): 3; Revised Child Anxiety and Depression Scale (RCADS): 1; Strengths and Difficulties Questionnaire (SDQ): 1; Longitudinal Interval Follow‐up Evaluation (LIFE): 2; Structured Clinical Interview for DSM (SCID)/Structured Clinical Interview for DSM Non Patient (SCID‐NP): 3; Social Phobia Inventory (SPIN): 1; Clinician rated diagnosis: 1; Diagnostic Interview Schedule III Revised (DIS‐III‐R): 2; Diagnostic Interview for Children and Adolescents‐Revised (DICA‐R): 1; Brief Symptom Inventory (BSI): 1; Health and Behaviour Checklists: 1; Substance Use Risk Profile Scale (SURPS): 3; Screen for Child Anxiety Related Disorders (SCARED): 1; Rutter Scales: 1; Revised Children's Manifest Anxiety Scale (RCMAS): 1; Mini‐Mood and Anxiety Symptom Questionnaire (MASQ): 1; Multidimensional Peer Nomination Inventory (MPNI): 1; Anxiety Sensitivity Index (ASI): 1; Revised Behaviour Problem Checklist (RBPC): 1; Symptom Check List (SCL‐5): 1; Brief Child and Family Phone Interview (BCFPI): 1; Anxiousness (based on the symptom clusters in DSM‐5): 1; Multidimensional Anxiety Scale for Children (MASC‐ 10): 1; State–Trait Personality Inventory (STPI): 1; and 2 researcher‐constructed measures.

Alcohol measures: Diagnostic Interview Schedule for Children (DISC): 2; Diagnostic Interview Schedule (DIS): 2; Munich‐Composite International Diagnostic Interview (M‐CIDI): 5; Longitudinal Interval Follow‐up Evaluation (LIFE): 4; Structured Clinical Interview for DSM (SCID)/Structured Clinical Interview for DSM Non Patient (SCID‐NP): 5; National Youth Survey (NYS): 3; Clinical Interview Schedule (CIS): 1; Youth Risk Behavior Surveillance System (YRBSS): 1; Adult Heath Survey: 1; Adolescent Health Survey: 1; Kiddie Schedule for Affective Disorders and Schizophrenia (K‐SADS): 3; Measures adapted from Questionnaire for the Alcohol, Health, and Behavior study: 1; Drinking and Drug History Questionnaire: 1; Diagnostic Interview for Children and Adolescents‐Revised (DICA‐R): 1; Composite International Diagnostic Interview: 3; CAGE Questionnaire (cut‐annoyed‐guilty‐eye): 1; Semi‐Structured Assessment for the Genetics of Alcoholism (SSAGA): 2; Youth Self‐Report (YSR): 1; Michigan Alcohol Screening Test (MAST): 1; Harmful Effects of Alcohol Scale: 1; Adolescent risk behaviour survey: 1; and 19 researcher‐constructed measures.

Full data extraction information can be found in Supporting information, Table S2 **.**

### Narrative synthesis

Below we present a summary of associations organized by alcohol outcome. There were 97 associations in total (see Tables 2, 3, 4).

**Table 2:** Number of positive, negative, equivocal and unclassifiable associations between an anxiety exposure and a drinking frequency/quantity outcome.

Anxiety phenotype	Number of studies	Negative	Weak negative	Equivocal	Weak positive	Positive	Unclassifiable
Generalized anxiety disorder	5	1 (20%)	0 (0%)	2 (40%)	0 (0%)	1 (20%)	1 (20%)
Internalizing disorders	12	2 (14%)	1 (7%)	5 (36%)	0 (0%)	1 (7%)	5 (36%)
Miscellaneous anxiety	9	1 (10%)	1 (10%)	1 (10%)	0 (0%)	3 (30%)	4 (40%)
Obsessive compulsive disorder	0	0 (0%)	0 (0%)	0 (0%)	0 (0%)	0 (0%)	0 (0%)
Panic disorder	1	0 (0%)	0 (0%)	0 (0%)	0 (0%)	1 (100%)	0 (0%)
Separation anxiety disorder	1	0 (0%)	0 (0%)	0 (0%)	1 (100%)	0 (0%)	0 (0%)
Social anxiety Disorder	5	2 (40%)	0 (0%)	0 (0%)	0 (0%)	2 (40%)	1 (20%)
Specific phobias	1	0 (0%)	0 (0%)	1 (100%)	0 (0%)	0 (0%)	0 (0%)
Total	27	6 (16%)	2 (5%)	9 (24%)	1 (3%)	8 (22%)	11 (30%)

Drinking frequency/quantity outcomes include: hazardous drinking, heavy drinking, drinking frequency, alcohol quantity and alcohol use.

Number of studies total = number of studies which reported an association between an anxiety exposure and a drinking frequency/quantity outcome. Note that some studies examined multiple anxiety disorders.

**Table 3:** Number of positive, negative, equivocal and unclassifiable associations between an anxiety exposure and a binge drinking outcome.

Anxiety phenotype	Number of studies	Negative	Weak negative	Equivocal	Weak positive	Positive	Unclassifiable
Generalized anxiety disorder	3	1 (33%)	0 (0%)	1 (33%)	1 (33%)	0 (0%)	0 (0%)
Internalizing disorders	4	0 (0%)	2 (40%)	1 (20%)	0 (0%)	0 (0%)	2 (40%)
Miscellaneous anxiety	3	0 (0%)	0 (0%)	0 (0%)	1 (25%)	0 (0%)	3 (75%)
Obsessive compulsive disorder	0	0 (0%)	0 (0%)	0 (0%)	0 (0%)	0 (0%)	0 (0%)
Panic disorder	0	0 (0%)	0 (0%)	0 (0%)	0 (0%)	0 (0%)	0 (0%)
Separation anxiety disorder	1	0 (0%)	0 (0%)	0 (0%)	1 (100%)	0 (0%)	0 (0%)
Social anxiety disorder	1	1 (100%)	0 (0%)	0 (0%)	0 (0%)	0 (0%)	0 (0%)
Specific phobias	0	0 (0%)	0 (0%)	0 (0%)	0 (0%)	0 (0%)	0 (0%)
Total	9	2 (14%)	2 (14%)	2 (14%)	3 (21%)	0 (0%)	5 (36%)

Binge drinking outcomes include: binge drinking, heavy episodic drinking and intoxication/drunkenness.

Number of studies total = number of studies which reported an association between an anxiety exposure and a binge drinking outcome. Note that some studies examined multiple anxiety disorders.

**Table 4:** Number of positive, negative, equivocal and unclassifiable associations between an anxiety exposure and an alcohol use disorder outcome.

Anxiety phenotype	Number of studies	Negative	Weak negative	Equivocal	Weak positive	Positive	Unclassifiable
Generalized anxiety disorder	6	1 (14%)	0 (0%)	5 (71%)	0 (0%)	0 (0%)	1 (14%)
Internalizing disorders	5	0 (0%)	1 (20%)	0 (0%)	0 (0%)	3 (60%)	1 (20%)
Miscellaneous anxiety	12	1 (8%)	0 (0%)	4 (31%)	0 (0%)	5 (38%)	3 (23%)
Obsessive compulsive disorder	2	0 (0%)	0 (0%)	0 (0%)	1 (50%)	0 (0%)	1 (50%)
Panic disorder	6	0 (0%)	0 (0%)	1 (17%)	1 (17%)	3 (50%)	1 (17%)
Separation anxiety disorder	2	0 (0%)	0 (0%)	1 (50%)	0 (0%)	1 (50%)	0 (0%)
Social anxiety disorder	7	1 (13%)	1 (13%)	2 (25%)	1 (13%)	3 (38%)	0 (0%)
Specific phobias	3	0 (0%)	0 (0%)	1 (33%)	1 (33%)	1 (33%)	0 (0%)
Total	26	3 (7%)	2 (4%)	14 (30%)	4 (9%)	16 (35%)	7 (15%)

Alcohol use disorder outcomes include: alcohol dependence, alcoholism, harmful drinking, alcohol use disorders, alcohol problems and alcohol abuse.

Number of studies total = number of studies which reported an association between an anxiety exposure and an alcohol use disorder outcome. Note that some studies examined multiple anxiety disorders.

#### Alcohol consumption (collectively)

Among all alcohol outcomes, there were 32 (33%) positive associations, 17 (18%) negative associations, 25 (26%) equivocal associations and 23 (24%) unclassifiable associations. There were more positive than negative associations for AUD (20 versus five), compared to drinking frequency/quantity (nine versus eight) and binge drinking (three versus four). Findings were robust to the removal of the 24 internalizing associations (where anxiety and depression could not be distinguished): 28 (38%) positive associations, 11 (15%) negative associations, 19 (26%) equivocal associations and 15 (21%) unclassifiable associations.

We explored whether the mixed findings were due to heterogeneity of anxiety. There were only positive associations (not negative) for OCD (one), panic disorder (five), separation anxiety (three) and specific phobias (two). There were more positive than negative associations for miscellaneous anxiety (nine versus three) and social anxiety (six versus five). There were more negative than positive associations for GAD (three versus two) and internalizing disorders (six versus four). There were equivocal associations for all anxiety disorders, except OCD. We also explored whether there were differences according to sample age. Of the seven associations where anxiety was measured in childhood, one was positive (14%), two were negative (29%) and four were equivocal (57%). Of the 87 associations where anxiety was measured in adolescence, 31 were positive (36%), 14 were negative (16%), 19 were equivocal (22%) and 23 were unclassifiable (26%). For three associations, developmental period was unclear.

#### Drinking frequency/quantity

There were 37 associations between anxiety and drinking frequency/quantity (see Table 2). Nine were positive associations. For all nine, anxiety was measured in adolescence only, and for eight (89%), drinking frequency/quantity was assessed less than 4 years later. Seven (78%) associations were statistically adjusted for gender and four (44%) were adjusted for other psychological disorders. Four (44%) were based on a sample size greater than 1000. There were eight negative associations. Five (63%) measured anxiety in adolescence only, and there was no pattern in length of follow‐up. Two (25%) associations were adjusted for gender and four (50%) were adjusted for other psychological disorders. Five (63%) were based on a sample size greater than 1000. There were nine equivocal associations. Five (56%) came from adolescent samples only, two (22%) came from samples which included young adults and two (22%) came from samples which included children. For six (67%) associations, drinking frequency/quantity was assessed less than 4 years later. Six (67%) associations were adjusted for gender and two (22%) were adjusted for other psychological disorders. Three (33%) were based on a sample size greater than 1000.

#### Binge drinking

There were 14 associations between anxiety and binge drinking (see Table 3). Three were positive associations. All three assessed anxiety in adolescence and measured alcohol use less than 4 years later. One (33%) adjusted for gender and another psychological disorder and one was based on a sample size greater than 1000. There were four negative associations. Two (50%) assessed anxiety in adolescence and two (50%) in childhood. One (25%) adjusted for gender and one (25%) adjusted for another psychological disorder. Three (75%) were based on a sample size greater than 1000. There were two equivocal associations. Both involved maternal reported anxiety and binge drinking was assessed in adolescence.

#### Alcohol use disorders

There were 46 associations between anxiety and AUD (see Table 4). Twenty were positive associations. Nineteen (95%) measured anxiety in adolescence and one (5%) measured anxiety in childhood. For 13 (65%) associations, AUD was assessed 10 or more years later. Sixteen (80%) were adjusted for gender and seven (35%) were adjusted for other psychological disorders. Eight (40%) were based on a sample size greater than 1000. There were five negative associations. All five assessed anxiety in adolescence and AUD was assessed more than 10 years later for two (40%) associations. Two (40%) associations were adjusted for gender and one (20%) was adjusted for other psychological disorders. One (20%) was based on a sample size greater than 1000.

There were 14 equivocal associations. Twelve (86%) related to anxiety in adolescence and two (14%) in childhood. For eight (57%) associations, AUD was assessed more than 10 years later. Eight (57%) associations were adjusted for gender and five (36%) were adjusted for other psychological disorders. Four (29%) were based on sample sizes greater than 1000.

### Meta‐analysis

**Figure 2:**
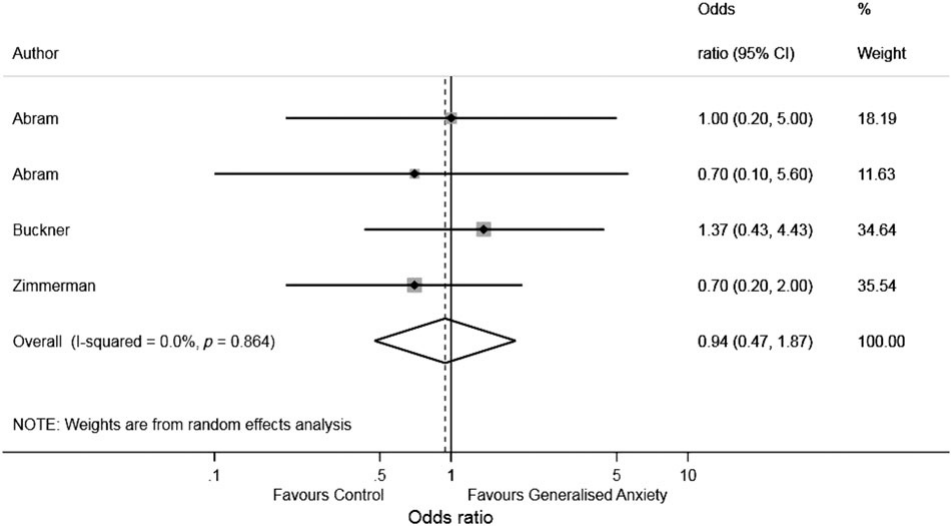
Forest plot of associations between generalized anxiety and alcohol use disorder

There was no clear evidence that GAD is associated with later AUD [odds ratio (OR = 0.94, 95% CI = 0.47–1.87, *I* ^2^ = 0%]. A forest plot summarizing the individual study estimates and pooled estimate is shown in Fig. 2.

## Discussion

### Main findings

Our meta‐analysis revealed no clear evidence of an association between GAD and AUD. In our systematic narrative review, one‐third of associations were positive (i.e. anxiety was prospectively associated with greater alcohol consumption), supporting the self‐medication hypothesis. However, approximately one‐fifth of associations were negative (i.e. anxiety was prospectively associated with lower alcohol consumption) and a quarter of associations were equivocal.

When separating associations by alcohol outcome, there was some evidence for a positive relationship between anxiety and AUD. This appeared to be driven by all anxiety categories except GAD. There were no positive associations between GAD and AUD compared, for example, to panic disorder, where there were four positive associations. This may be explained by differences in symptoms. People with panic disorder experience higher sympathetic nervous system arousal (e.g. racing heart, shortness of breath) than people with GAD 75, 76. There is also evidence that physiological anxiety symptoms are positively associated with alcohol dependence, whereas cognitive symptoms are negatively associated 12. Associations of anxiety with drinking frequency/quantity and binge drinking were unclear and inconsistent; there were a similar number of positive, negative and equivocal results.

There were no negative associations for OCD, panic disorder, separation anxiety and specific phobias. There were positive and negative associations for miscellaneous anxiety, social anxiety, GAD and internalizing disorders. Other sources of between‐study heterogeneity, including developmental period of anxiety, length of follow‐up, confounders adjusted for and sample size, did not appear to account for inconsistent findings. It was difficult to compare associations for child versus adolescent anxiety because of the imbalance in quantity (seven versus 87 associations). This arose because we avoided counting non‐independent associations. If studies reported several associations at different ages, we selected adolescence because that was the key developmental period for our synthesis and measurement bias was less likely.

### Strengths and limitations

To our knowledge, this is the largest systematic review investigating prospective associations between different anxiety exposures and later alcohol use outcomes. There are limitations. First, we only included English language publications which may have produced bias. Studies with significant positive results are more likely to be published in English language journals 77, therefore we may have missed relevant studies, particularly those with null findings. Secondly, our approach to coding the evidence resulted in several unclassifiable associations, as many studies did not report exact *P*‐values or CIs. However, coding associations by the strength of evidence was considered more accurate than using an arbitrary (e.g. *P* < 0.05) threshold, despite loss of data. Thirdly, some studies with more sophisticated statistical models were excluded, as they did not report prospective associations. Fourthly, although we restricted to prospective studies to elucidate the temporal sequence of anxiety and alcohol use, we cannot infer causality from observational studies. For example, several studies did not adjust for important potential confounders (or did not report this), and there may be residual confounding. Fifthly, some studies may have been underpowered to detect an association due to small samples. Finally, one limitation of our review, and the literature in general, may be the use of broad measures of internalizing behaviour. We cannot determine what proportion of internalizing measures assess depression rather than anxiety without additional specific measures, which were unavailable in some studies. Given that depression can be a more consistent predictor of alcohol use than anxiety 25, the use of internalizing measures as a proxy for anxiety may contribute to misclassification or measurement bias. We included internalizing disorders in our search strategy to ensure comprehensiveness and because the term is often used when referring to symptoms in children. The overall findings remained unchanged when we excluded internalizing associations.

### Other evidence

Overall, a clear association between anxiety and alcohol use was not evident, consistent with previous reviews 24, 25. When distinguishing between alcohol outcomes, anxiety was generally positively associated with AUD, supporting a previous meta‐analysis which found that social anxiety was associated with alcohol‐related problems 9. However, the authors also found that social anxiety was negatively associated with general use, whereas we found positive, negative and equivocal associations between anxiety and drinking frequency/quantity.

### Future directions and implications

There are different possible explanations for our findings. To assess the causality of observed associations between anxiety and AUD, future research should employ study designs which eliminate confounding and reverse causation, such as Mendelian randomization 78. Alternatively, there may be no causal relationship between anxiety disorders and AUD. The common‐factor model suggests that third variables (genetic or environmental) account for the comorbidity between these disorders 79. More research is needed to identify which variables attenuate or eliminate associations between anxiety and AUD. In addition, we did not include studies that investigated reverse temporal associations; greater alcohol use may increase susceptibility to anxiety disorders 10. These pathways are also important. Future systematic reviews which examine associations between alcohol use and subsequent anxiety are required to help elucidate temporal order and the validity of theoretical models.

We did not find compelling evidence of a relationship between anxiety and drinking frequency/quantity or binge drinking. However, absence of evidence is not evidence of absence. Firstly, some studies had methodological limitations, which may have led to Type 2 errors. Better‐quality studies, which are adequately powered and adjust for relevant confounders, would help to determine whether or not there is a genuine association. Secondly, the evidence may be equivocal, which suggests that any association is likely to be weak or context‐dependent. Thirdly, studies in the narrative synthesis may have been too heterogeneous to provide clear combined evidence, a concern raised by other researchers 25. Future meta‐analyses with a greater number of combinable studies would be informative, improving objectivity, power and precision. However, this will not be possible unless the relationship is investigated more consistently. Specifically, consistent types and measurements of anxiety and alcohol use, as well as full reporting of statistical information (e.g. exact *P*‐values and CIs), would facilitate future quantitative syntheses and meta‐analyses.

It may be important for future studies to distinguish between anxiety symptoms. For example, Stewart and colleagues 80 found that fear of negative evaluation was positively associated with drinking problems, whereas social avoidance and distress were negatively associated with drinking frequency. This suggests that anxiety disorders are complex and multi‐dimensional, and different associations with alcohol use within anxiety disorders should be explored. Examination of potential moderating variables such as gender, age, alcohol expectancies, drinking motives and stressful events may also help to explain discrepant findings. Large cohort studies which compare data at the group level cannot capture subtle dynamic differences in symptoms and behaviour, which may explain inconsistent findings. Future research could therefore utilize more sensitive methodological approaches which account for these complexities. Ecological momentary assessment studies, with repeated real‐time assessments of anxiety and alcohol use, may be a more nuanced approach to capturing the relationship and within‐participant variation. Understanding individual differences in anxiety–alcohol comorbidity could lead to improvements in personalized interventions.

In summary, we found some evidence that child and adolescent anxiety was positively associated with later AUD, whereas the relationship with drinking frequency/quantity and binge drinking was inconsistent. Study characteristics did not appear to account for inconsistent findings. A lack of clear evidence may be due to between‐study heterogeneity or weaknesses of individual studies. We discuss possible directions for future research to further investigate the relationship between anxiety and alcohol use. It is important to establish which anxious individuals consume more alcohol and develop AUD, in order to develop targeted interventions.

### Declarations of interests

None.

## Supporting information


**Table S1** Excluded full‐text articles with reasons.
**Table S2** Characteristics of included studies (complete data extraction).
