# Supplementary material for: Associations of child and adolescent anxiety with later alcohol use and disorders: a systematic review and meta‐analysis of prospective cohort studies
Source: Addiction. 2019 Mar 19;114(6):968–82. doi: 10.1111/add.14575 (PMC6563455; doi:10.1111/add.14575)
Supplement: Supplementary file 1 — Table S1 Excluded full‐text articles with reasons. Table S2 Characteristics of included studies (complete data extraction). [file ADD-114-968-s001.docx]

**Supplementary Material**

**Scopus example of search strategy.**

(TITLE-ABS-KEY ("anxi*" OR "internali?ing" OR "phobi*" OR "*phobia" OR "panic" OR "OCD" OR "obsessive-compulsive" OR "PTSD" OR "post-traumatic stress disorder") AND TITLE-ABS-KEY ("alcohol*" OR "ethanol" OR "drink*") AND TITLE-ABS-KEY ("longitudinal" OR "prospective" OR "cohort" OR "trajector*" OR "wave*") AND NOT TITLE-ABS-KEY ("rodent*" OR "mice" OR "mouse" OR "rat" OR “rats”)).

**Table S1. Excluded full-text articles with reasons.**

| **First Author** | **Year** | **Title** | **Reason for Exclusion** |
| --- | --- | --- | --- |
| Gerbino | 2017 | Protective and risk factors of alcohol and drug abuse in adolescence | Not English language |
| Hinckers | 2005 | Alcohol consumption in adolescence - social and individual influential factors | Not English language |
| Andréasson | 1992 | Antecedents and covariates of high alcohol consumption in young men | Cross-sectional / follow-up < 6 months |
| Birrell | 2005 | Anxiety disorders and first alcohol use in the general population. Findings from a nationally representative sample | Cross-sectional / follow-up < 6 months |
| Donbaek | 2014 | Post-traumatic stress disorder symptom clusters predicting substance abuse in adolescents | Cross-sectional / follow-up < 6 months |
| Goldstein | 2012 | Coping motives as moderators of the relationship between emotional distress and alcohol problems in a sample of adolescents involved with child welfare | Cross-sectional / follow-up < 6 months |
| Goodwin | 2004 | Association between anxiety disorders and substance use disorders among young persons: Results of a 21-year longitudinal study | Cross-sectional / follow-up < 6 months |
| Sartor | 2007 | The role of childhood risk factors in initiation of alcohol use and progression to alcohol dependence | Cross-sectional / follow-up < 6 months |
| Delfabbro | 2016 | Mid-adolescent predictors of adult drinking levels in early adulthood and gender differences: Longitudinal analyses based on the South Australian School Leavers Study | No anxiety exposure |
| Edwards | 2016 | A prospective longitudinal model predicting early adult alcohol problems: evidence for a robust externalizing pathway | No anxiety exposure |
| Buckner | 2009 | Understanding social anxiety as a risk for alcohol use disorders: Fear of scrutiny, not social interaction fears, prospectively predicts alcohol use disorders | Anxiety not in childhood/adolescence |
| Buckner | 2009 | Social anxiety disorder as a risk factor for alcohol use disorders: A prospective examination of parental and peer influences | Anxiety not in childhood/adolescence |
| Cheng | 2013 | Correlates of adult binge drinking: Evidence from a British cohort | Anxiety not in childhood/adolescence |
| Haller | 2014 | Risk pathways among traumatic stress, posttraumatic stress disorder symptoms, and alcohol and drug problems: A test of four hypotheses | Anxiety not in childhood/adolescence |
| Swendsen | 2010 | Mental disorders as risk factors for substance use, abuse and dependence: Results from the 10-year follow-up of the National Comorbidity Survey | Anxiety not in childhood/adolescence |
| Zatzick | 2002 | Posttraumatic stress, problem drinking, and functional outcomes after injury | Anxiety not in childhood/adolescence |
| Wu | 2010 | Trauma, posttraumatic stress symptoms, and alcohol-use initiation in children | PTSD exposure |
| Cisler | 2011 | PTSD symptoms, potentially traumatic event exposure, and binge drinking: A prospective study with a national sample of adolescents | PTSD exposure |
| Goldstein | 2011 | The relationship between post-traumatic stress symptoms and substance use among adolescents involved with child welfare: Implications for emerging adulthood | PTSD exposure |
| Alamian | 2012 | Individual and social determinants of multiple chronic disease behavioral risk factors among youth | No alcohol outcome |
| Bardone | 1998 | Adult physical health outcomes of adolescent girls with conduct disorder, depression, and anxiety | No alcohol outcome |
| Barnea | 1992 | Personality, cognitive, and interpersonal factors in adolescent substance use: A longitudinal test of an integrative model | No alcohol outcome |
| Brook | 2012 | Individuality and contextual Influences on drug dependence: A 15-year prospective longitudinal study of adolescents from Harlem | No alcohol outcome |
| Lewinsohn | 2008 | Separation anxiety disorder in childhood as a risk factor for future mental illness | No alcohol outcome |
| Lillehoj | 2004 | Internalizing, social competence, and substance initiation: influence of gender moderation and a preventive intervention | No alcohol outcome |
| Loeber | 1999 | Developmental aspects of delinquency and internalizing problems and their association with persistent juvenile substance use between ages 7 and 18 | No alcohol outcome |
| Siebenbruner | 2006 | Developmental antecedents of late adolescence substance use patterns | No alcohol outcome |
| Sung | 2004 | Effects of age at first substance use and psychiatric comorbidity on the development of substance use disorders | No alcohol outcome |
| Teichman | 1989 | Personality and substance use among adolescents: A longitudinal study | No alcohol outcome |
| Zehe | 2013 | Social and generalized anxiety symptoms and alcohol and cigarette use in early adolescence: The moderating role of perceived peer norms | No alcohol outcome |
| Cerdá | 2013 | Cumulative and recent psychiatric symptoms as predictors of substance use onset: Does timing matter? | Alcohol initiation was only outcome |
| Donovan | 2011 | Childhood risk factors for early-onset drinking | Alcohol initiation was only outcome |
| Fite | 2006 | Childhood behavior problems and peer selection and socialization: Risk for adolescent alcohol use | Alcohol initiation was only outcome |
| Geels | 2013 | Developmental prediction model for early alcohol initiation in Dutch adolescents | Alcohol initiation was only outcome |
| Kaplow | 2001 | The prospective relation between dimensions of anxiety and the initiation of adolescent alcohol use | Alcohol initiation was only outcome |
| McCarty | 2012 | Emotional health predictors of substance use initiation during middle school | Alcohol initiation was only outcome |
| Farmer | 2013 | Aggregation of lifetime Axis I psychiatric disorders through age 30: Incidence, predictors, and associated psychosocial outcomes | No association between anxiety and alcohol use |
| Skeer | 2009 | A prospective study of familial conflict, psychological stress, and the development of substance use disorders in adolescence | No association between anxiety and alcohol use |
| Costello | 1999 | Development of psychiatric comorbidity with substance abuse in adolescents: Effects of timing and sex | Concurrent or retrospective analyses, despite prospective data |
| Black | 2015 | Course of alcohol symptoms and social anxiety disorder from adolescence to young adulthood | Concurrent or retrospective analyses, despite prospective data |
| Colder | 2017 | Internalizing and externalizing problem behavior: A test of a latent variable interaction predicting a two-part growth model of adolescent substance use | Concurrent or retrospective analyses, despite prospective data |
| Pape | 2016 | Associations between emotional distress and heavy drinking among young people: A longitudinal study | Concurrent or retrospective analyses, despite prospective data |
| Stice | 1998 | A longitudinal grouping analysis of adolescent substance use escalation and de-escalation | Concurrent or retrospective analyses, despite prospective data |
| Wennberg | 2002 | Psychosocial characteristics at age 10; differentiating between adult alcohol use pathways: A prospective longitudinal study | Concurrent or retrospective analyses, despite prospective data |

**Table S2. Characteristics of included studies (complete data extraction).**

| **Study** | **Sample & Country** | **% Male** | **Anxiety**  **Type (Measure)**  **Age**  **Respondent** | **Alcohol Use**  **Type (Measure)**  **Age**  **Respondent** | **Follow-Up Time** | **Analysis Method & Results Summary** | **Confounders** | **Sample Size** | **Count Result**  **(Y/N)** | **Evidence**  **(N/WN/E/WP/P/U)** |
| --- | --- | --- | --- | --- | --- | --- | --- | --- | --- | --- |
| (1) | Youth at a juvenile detention centre, USA | 64 | Generalised anxiety disorder (GAD) (DISC-2.3)  10-19 (median 15)  Self-report | Alcohol use disorder (AUD) (DISC-IV)  15-25 (median 20)  Self-report | 5 years | ***Logistic regression:***  GAD 🡪 AUD  OR [95% CI]: Males 1.0 [0.2 to 5.0], p > .05  OR [95% CI]: Females 0.7 [0.1 to 5.6], p > .05 | Baseline AUD | 1504 (960 M, 544 F) | Y  Y | E  E |
| (2) | Early Developmental Stages of Psychopathology Study, Germany | 33 | Panic attacks (DSM-IV-TR M-CIDI)  14-24 (median 19)  Self-report | Alcohol use disorder (DIA-X/M-CIDI)  21-34  Self-report | 10 years | ***Logistic regression:***  Panic attacks 🡪 AUD  OR [95% CI]: 2.40 [1.12 to 5.16], p = .025 | Sex, age, and agoraphobia, GAD, social phobia, major depressive disorder, dysthymia, substance use disorder at T1 | 122 | Y | P |
| (3) | Early Developmental Stages of Psychopathology Study, Germany | 51 | Specific phobias (DIA-X/M-CIDI)  14-24  Self-report | Alcohol use, alcohol abuse, alcohol dependence (DIA-X/M-CIDI)  1.6, 3.5, and 8.2 years later  Self-report | 10 years | ***Cox regression:***  Specific phobia 🡪 alcohol abuse  HR [95% CI]: 1.07 [0.8 to 1.4], p = .574  Specific phobia 🡪 alcohol dependence  HR [95% CI]: 1.62 [1.1 to 2.3], p = .007  Social phobia 🡪 alcohol abuse  HR [95% CI]: 1.26 [0.8 to 1.8], p = .187  Social phobia 🡪 alcohol dependence  HR [95% CI]: 1.39 [0.8 to 2.2], p = .155  Panic attacks 🡪 alcohol abuse  HR [95% CI]: 1.34 [0.9 to 1.9], p = .084  Panic attacks 🡪 alcohol dependence  HR [95% CI]: 1.35 [0.8 to 2.1], p = .158  Any anxiety disorder 🡪 alcohol abuse  HR [95% CI]: 1.07 [0.8 to 1.3], p = .472  Any anxiety disorder 🡪 alcohol dependence  HR [95% CI]: 1.59 [1.1 to 2.2], p = .003 | Age, gender, any mood disorder, non-alcohol substance use disorders (SUD) (nicotine, cannabis, other illegal drugs), externalising disorders | 2929 | N  Y  N  Y  N  Y  N  Y | P  WP  WP  P |
| (4) | Early Developmental Stages of Psychopathology Study, Germany | 49 | Separation anxiety disorder (SAD): subthreshold, threshold (M-CIDI)  14-17  Self-report | Alcohol abuse, alcohol dependence (M-CIDI)  20 and 42 months later  Self-report | 20 months; 42 months | ***Cox regression:***  Threshold SAD (vs. no) 🡪 alcohol abuse  HR [95% CI]: 0.5 [0.0 to 2.8], no p value  Threshold SAD (vs. no) 🡪 alcohol dependence  HR [95% CI]: 4.7 [1.7 to 12.4], no p value  Subthreshold SAD (vs. no) 🡪 alcohol abuse  HR [95% CI]: 0.9 [0.5 to 1.6], no p value  Subthreshold SAD (vs. no) 🡪 alcohol dependence  HR [95% CI]: 2.1 [1.1 to 4.1], no p value  ***Logistic regression:***  SAD 🡪 alcohol dependence  OR [95% CI]: 3.3 [1.06 to 10.2], no p value | Age and sex | 1090 | N  N  N  N  Y | P |
| (5) | Oregon Adolescent Depression Project, USA | 41 | Social anxiety, generalised anxiety, separation anxiety, panic disorder, obsessive-compulsive disorder (OCD), overanxious disorder, specific phobia (K-SADS, K-SADS-P)  16  Self-report | Alcohol abuse, alcohol dependence (LIFE, SCID-4)  30  Self-report | 14 years | ***Logistic regression:***  OCD 🡪 alcohol abuse  OR [95% CI]: 2.44 [0.41 to 14.72], p > .05  OCD 🡪 alcohol dependence  OR [95% CI]: 5.18 [0.86 to 31.26], p > .05  Over-anxious disorder 🡪 alcohol abuse  OR [95% CI]: 0.28 [0.04 to 2.12], p > .05  Over-anxious disorder 🡪 alcohol dependence  OR [95% CI]: 1.37 [0.43 to 4.43], p > .05  Specific phobia 🡪 alcohol abuse  OR [95% CI]: 0.78 [0.22 to 2.73], p > .05  Specific phobia 🡪 alcohol dependence  OR [95% CI]: 1.89 [0.69 to 5.18], p > .05  Separation anxiety disorder 🡪 alcohol abuse  OR [95% CI]: 0.56 [0.23 to 1.35], p > .05  Separation anxiety disorder 🡪alcohol dependence  OR [95% CI]: 0.87 [0.41 to 1.85], p > .05  Social anxiety disorder 🡪 alcohol abuse  OR [95% CI]: 0.48 [.11 to 2.11], p > .05  Social anxiety disorder 🡪 alcohol dependence  OR [95% CI]: 3.98 [1.51 to 10.47], p < .01  Panic disorder 🡪 alcohol abuse  OR [95% CI]: 0.52 [0.06 to 4.23], p > .05  Panic disorder 🡪 alcohol dependence  OR [95% CI]: 5.82 [1.38 to 24.57], p < .05  ***Hierarchical logistic regression:***  Social anxiety disorder 🡪 alcohol dependence  OR [95% CI]: 4.47 [1.48 to 13.45], p <.01  Panic disorder 🡪 alcohol dependence  OR [95% CI]: 2.36 [0.25 to 21.96], p > .05 | Gender  T1 AUD, mood disorder, conduct disorder, gender | 816 | N  Y  N  Y  N  Y  N  Y  N  Y  N  Y  N  N | WP  E  WP  E  P  P |
| (6) | Pittsburgh Youth Study, USA | 100 | Anxiety problems (CBCL, TRF, YSR, YASR)  13-19 (annual or semi-annual)  Caregiver-, teacher-, self-report | Alcohol frequency, alcohol quantity (Substance Use Scale from NYS)  13–19 (semi-annual)  Self-report | 6 years | ***Quasi-Poisson models:***  Changes in anxiety 🡪 changes in frequency  Rate ratio [95% CI]: 0.98 [0.95 to 1.02], no p value  Changes in anxiety 🡪 changes in quantity  Rate ratio [95% CI]: 0.99 [0.97 to 1.00], no p value | Frequency model: age, prior marijuana frequency and alcohol quantity. Quantity model: additionally, conduct problems, peer delinquency, and frequency (instead of quantity) | 487 | Y  N | WN |
| (7) | Taiwan Aboriginal Study Project, Taiwan | 30 | Anxiety disorders (Chinese CIS)  15-24  Self-report | Time to onset of alcoholism (Chinese CIS)  4 years later  Self-report | 4 years | ***Cox proportional hazards regression:***  Anxiety disorders 🡪 alcoholism  Relative risk [95% CI]: 0.65 [0.20 to 2.10], p = .47 | None/no information | 164 | Y | E |
| (8) | From a longitudinal study of adolescent substance use, USA | 45 | Internalising problems (YSR)  11-13  Self-report | Alcohol use (NYS)  12-16  Self-report | 3 years | ***Structural equation model:***  Internalising symptoms 🡪 alcohol use  Beta (standardised) = − 0.09, p >.10 | Gender and age | 367 | Y | E |
| (9) | From a longitudinal study of HIV-related risk behaviours, USA | 56 | Social phobia (SP) (RCADS)  11, 12, 13, 14, 15  Self-report | Alcohol use (modiﬁed version of YRBSS)  11, 12, 13, 14, 15  Self-report | 1-4 years | ***Generalized estimating equations (GEEs):***  Social phobia baseline 🡪 alcohol consumption  OR [95% CI]: 1.06 [1.00 to 1.12], p = .051  Social phobia prior year 🡪 alcohol consumption  OR [95% CI]: 1.06 [1.01 to 1.11], p = .03 | Baseline age, gender, MDD symptoms, linear effect of time, interaction between baseline SP symptoms and time, interaction between MDD symptoms and time | 277 | N  Y | P |
| (10) | Avon Longitudinal Study of Parents and Children, UK | 51 | Internalising symptom trajectories (SDQ)  3, 6, 8, 9, 11  Maternal-report | Whole drink, drank without parental permission, ever binge, number of whole drinks in past 6 months  13  Self-report | Trajectories (max 8 years) | ***Growth mixture modelling:***  Internalising symptoms 🡪 number of drinks  Standardised parameter estimates [95% CI]:  Persistently high class (vs stable low)  −.92 [−1.04 to −.80], p < .01  Mid-childhood increase class (vs stable low)  −.66 [−1.01 to −.31], p < .01  High to low class (vs stable low)  −.78 [−1.07 to −.48], p < .01  Low to high class (vs stable low)  1.04 [−.42 to 0.63], p = .70  Internalising symptoms 🡪 ever binge drinking  Low to high (vs. stable low)  OR [95% CI]: .75 [.58 to .99], p = .04  Mid-childhood increase (vs. stable low)  OR [95% CI]: .72 [.57 to .91], p = < .01  Persistently high (vs. stable low)  OR [95% CI]: .94 [.65 to 1.35], p = .72  High to low (vs. stable low)  OR [95% CI]: .88 [.68 to 1.15], p = .36  Internalising 🡪 whole drink past 6 months  Persistently high  OR [95% CI]: .83 [.61 to 1.12], p = .22  Low to high  OR [95% CI]: .83 [.67 to 1.02], p = .08  Mid childhood increase  OR [95% CI]: .87 [.73 to 1.04], p = .12  High to low  OR [95% CI]: .77 [.62 to .95], p = .02  Internalising 🡪 drank without permission  Persistently high  OR [95% CI]: .87 [.61 to 1.24], p = .44  Low to high  OR [95% CI]: .86 [.67 to 1.09], p = .21  Mid-childhood increase  OR [95% CI]: 1.04 [.85 to 1.27], p = .71  High to low  OR [95% CI]: .75 [.58 to .97], p = .03 | Sex, maternal depression, income, and correlations among predictive variables | 11157 | Y  N  N  N  N  N  Y  N  N  N  N  N  N  N  N  N | N  E |
| (11) | Minnesota Longitudinal Study of Parents and Children, USA | 53 | Internalising behaviour (TRF of Child Behavior Checklist)  9  Teacher-report | Abstainers, moderate drinkers, heavy drinkers, and alcohol use disorder (Adult Health Survey)  19, 23, 26, 28  Self-report | 10-19 years | ***Multinomial logistic regression:***  M: Internalising 🡪 abstainers vs heavy users (19)  OR [95% CI]: 1.05 [0.98 to 1.14], p > .05  M: Internalising 🡪 moderate vs heavy users (19)  OR [95% CI]: 1.05 [0.99 to 1.12], p > .05  M: Internalising 🡪 abstainers vs heavy users (23)  OR [95% CI]: 1.00 [0.87 to 1.14], p > .05  M: Internalising 🡪 moderate vs heavy users (23)  OR [95% CI]: 0.97 [0.91 to 1.03], p > .05  M: Internalising 🡪 abstainers vs heavy users (26)  OR [95% CI]: 0.99 [0.90 to 1.10], p > .05  M: Internalising 🡪 moderate vs heavy users (28)  OR [95% CI]: 1.01 [0.95 to 1.07], p > .05  F: Internalising 🡪 abstainers vs heavy users (19)  OR [95% CI]: 0.95 [0.87 to 1.04], p > .05  F: Internalising 🡪 moderate vs heavy users (19)  OR [95% CI]: 0.99 [0.93 to 1.05], p > .05  F: Internalising 🡪 abstainers vs heavy users (23) OR [95% CI]: 0.82 [0.67 to 1.01], p > .05  F: Internalising 🡪 moderate vs heavy users (23)  OR [95% CI]: 0.97 [0.92 to 1.04], p > .05  F: Internalising 🡪 abstainers vs heavy users (26) OR [95% CI]: 0.99 [0.91 to 1.09], p > .05  F: Internalising 🡪 moderate vs heavy users (28)  OR [95% CI]: 1.00 [0.93 to 1.07], p > .05 | No information | 158-170 | N  N  N  N  Y  N  N  N  N  N  Y  N | E  E |
| (12) | Minnesota Longitudinal Study of Parents and Children, USA | 55 | Internalising symptoms (TRF, YSR)  7, 9, 12, 16  Teacher-report, self-report | Frequency and quantity of alcohol use (Adolescent Health Survey)  16  Self-report | 9 years max | ***Path models from developmental cascade modelling:***  Internalising symptoms 🡪 alcohol use  Unstandardised parameter estimates [95% CI]  .01 [−.03 to .04], p > .05  Internalising symptoms 🡪 level of use [95% CI]  −.01 [−.03 to .00] p > .05 | Gender, socioeconomic status, mother’s age at child’s birth, child’s minority status | 191 | N  Y | E |
| (13) | Oregon Adolescent Depression Project, USA | 41 | Anxiety disorders (K-SADS, LIFE, SCID)  Assessed 16, 17, 24, 30. Childhood (before 11), adolescent (after 11)  Self-report | Alcohol use disorder (K-SADS, LIFE, SCID)  24, 30  Self-report | 14-19 years | ***Binomial distribution with logit link function:***  Childhood anxiety 🡪 alcohol use disorder  OR [95% CI]: 0.93 [0.74 to 1.16], p > .0025  Adolescent anxiety 🡪 alcohol use disorder  OR [95% CI]: 1.29 [1.15 to 1.43], p < .0025 | Gender, MDD, AUD, SUD, and disruptive disorder before age 19 | 816 | N  Y | P |
| (14) | Oregon Adolescent Depression Project, USA | 38 (No AUD);  55 (AUD) | Anxiety disorders (AD) (K-SADS, LIFE, SCID-NP)  Assessed 16, 17, 24, 30. Childhood (8-12), early-to-middle adolescence (13-17), late adolescence (18-20), early adulthood (21-30)  Self-report | Alcohol use disorder (DSM III-R, DSM-IV, K-SADS, LIFE, SCID-NP)  Assessed 16, 17, 24, 30. Childhood (8-12), early-to-middle adolescence (13-17), late adolescence (18-20), early adulthood (21-30)  Self-report | 14 years | ***Cox proportional hazard modelling***  AD in childhood 🡪 early-to-middle adolescent AUD  HR [95% CI]: 1.38 [0.67 to 2.87], p > .05  AD in early-to-middle adolescence 🡪 late adolescent AUD  HR [95% CI]: 1.53 [0.72 to 3.22], p > .05  AD in childhood 🡪 late adolescent AUD onset  HR [95% CI]: 1.58 [0.70 to 3.55], p > .05  AD in late adolescence 🡪 early adult AUD onset  HR [95% CI]: 0.88 [0.43 to 1.82], p > .05  AD in early-to-middle adolescence 🡪 early adult AUD onset  HR [95% CI]: 1.71 [0.93 to 3.15], p > .05  AD in childhood 🡪 early adult AUD onset  HR [95% CI]: 1.15 [0.58 to 2.25], p > .05 | Gender, race/ethnicity, puberty onset, repeating a grade before age 12, at T1: dual vs single parent household, at least one parent completed college, mean age of heads of household; number of older siblings, externalising disorders | 641 | N  N  N  Y  N  N | E |
| (15) | Adolescent Mental Health Cohort Study, Finland | 44 | General anxiety (1 item), social phobia (SPIN)  15-16  Self-report | Frequent alcohol use, frequent drunkenness  17-18  Self-report | 2 years | ***Logistic regression:***  General anxiety 🡪 frequent alcohol use  IOR [95% CI]: 2.4 [1.2 to 4.8]  Social phobia 🡪 frequent alcohol use  IOR [95% CI]: 0.5 [0.3 to 0.8]  General anxiety 🡪 frequent drunkenness  IOR [95% CI]: 1.5 [0.6 to 3.9]  Social phobia 🡪 frequent drunkenness  IOR [95% CI]: 0.3 [0.1 to 0.8] | Sex, family structure, parental education, depression | 2070 | Y  Y  Y  Y | P  N  WP  N |
| (16) | The British Child and Adolescent Mental Health Surveys, UK | 52 | Internalising symptoms (SDQ, DAWBA), internalising disorder (clinical diagnosis)  11-12, 13-14, 15-16  Clinician-, parent-, teacher-report | Frequent alcohol consumption (different item for each group)  3 years later  Self-report | 3 years | ***Logistic regression:***  Internalising (SDQ) 🡪 frequent consumption  OR [95% CI]: 0.96 [0.91 to 1.02], p > .05  Internalising (DAWBA) 🡪 frequent consumption  OR [95% CI]: 0.93 [0.75 to 1.16], p > .05  Internalising disorder 🡪 frequent consumption  OR [95% CI]: 1.01 [0.52 to 1.95], p > .05 | Gender, age, baseline substance use, smoking, alcohol use, cannabis use and other illicit drug use, survey year, country, ethnic group, parent education, housing tenure, family type | 3607 | N  N  Y | E |
| (17) | Early Developmental Stages of Psychopathology Study, Germany | No info | Panic attacks (M-CIDI)  14-24  Self-report | Alcohol use disorder (M-CIDI)  14-25 and 34-50 months later  Self-report | 14-25 months; 34-50 months | ***Multiple logistic regression:***  Panic attacks 🡪 alcohol use disorder  OR [95% CI]: 2.4 [1.2 to 5.1], p < .05 | Age, gender, and other mental disorders | 2548 | Y | P |
| (18) | Oregon Adolescent Depression Project, USA | 44 (no anxiety); 30 (anxiety) | Anxiety disorders (K-SADS)  16  Self-report | Alcohol use disorder (K-SADS, LIFE)  16, 17, 24, 30  Self-report | 1-14 years | ***Cox proportional hazards models:***  Anxiety disorders 🡪 time to develop an AUD  HR [95% CI]: 1.07 [0.87 to 1.33], p = .51 (maternal support)  HR [95% CI]: 1.13 [0.89 to 1.44], p = .30 (paternal support) | Gender, parental education, number in household, whether biological parent, birth order, lifetime MDD or externalising disorder, coping skills | 817 | Y  N | E |
| (19) | From a longitudinal study of familial alcoholism, USA | 62 | Internalising symptoms (CBCL, CDIS-III-R)  11-15  Self-report | Alcohol problems (from Sher’s 1987 questionnaire)  25  Self-report | 12 years | ***Correlations:***  Internalising symptoms 🡪 alcohol problems  -.05, p >.10  ***Path analyses:***  No direct paths between adolescent internalising symptoms and alcohol problems. No numbers reported. | None for this analysis | 166 | N  Y | U |
| (20) | Seattle Social Development Project, USA | 50 | Behavioural inhibition/trait anxiety (CBCL)  14-15  Self-report | Alcohol abuse and alcohol dependence (DISC)  27  Self-report | 13 years | ***Multivariate linear regression:***  Behavioural inhibition/anxiety 🡪 alcohol abuse  Beta (standardised): 0.01, p = .86  Behavioural inhibition/anxiety 🡪 alcohol dependence  Beta (standardised): 0.04, p = .48 | Ethnicity, gender, poverty, past-month drinking at age 12 | 640 | N  Y | E |
| (21) | Michigan Longitudinal Study, USA | 69 | Distress/internalising symptoms (YSR of CBCL)  12-14  Self-report | Max number of drinks, heavy episodic drinking (Drinking and Drug History questionnaire)  18-20  Self-report | 6 years | ***Correlations:***  Distress/internalising 🡪 maximum number of drinks in a 24-hour period  .06, p > .01  Distress/internalising 🡪 heavy episodic drinking frequency  .04, p > .01 | None for this analysis | 1064 | N  Y | U |
| (22) | Project on Human Development in Chicago Neighbourhoods, USA | 51 | Internalising symptoms (YSR of CBCL)  12, 15, 18  Self-report | Alcohol Use (number of days drunk alcohol in the past month)  12, 15, 18  Self-report | 3 years | ***Cross-lagged structural equation models:***  Internalising symptoms age 12 and alcohol use age 15 (boys or girls) p > .05  Internalising symptoms age 15 and alcohol use age 18 (boys or girls) p > .05 | Gender, race/ethnicity, salary, education of primary caregivers | 724 | N  Y | U |
| (23) | Minnesota Twin Family Study, USA | 0 | Separation anxiety disorder, overanxious disorder (DICA-R)  10-12 (mean 11)  Self-report, maternal-report | Regular use, ever drunk, heavy drinking (DICA-R)  14  Self-report | 3 years | ***Generalized estimating equations (GEEs):***  Separation anxiety 🡪 regular use of alcohol 14  OR [95% CI]: 1.32 [0.71 to 2.47], p > .01  Separation anxiety 🡪 heavy drinking 14  OR [95% CI]: 1.36 [0.72 to 2.57], p > .01  Separation anxiety 🡪 getting drunk 14  OR [95% CI]: 1.57 [0.96 to 2.58], p > .01  Overanxious disorder 🡪 regular use of alcohol 14 OR [95% CI]: 0.81 [0.81 to 3.60], p > .01  Overanxious disorder 🡪 heavy drinking 14  OR [95% CI]: 1.00 [0.42 to 2.41], p > .01  Overanxious disorder 🡪 getting drunk 14  OR [95% CI]: 0.99 [0.64 to 1.54], p > .01 | No information | 699 twin  girls | N  Y  Y  N  Y  Y | WP  WP  E  E |
| (24) | From 24 secondary schools in London with personality risk for substance misuse, UK | 53 (drinker); 48 (non-drinker) | Anxiety (BSI)  13, 13.5, 14, 14.5  Self-report | Alcohol use (quantity x frequency)  13, 13.5, 14, 14.5    Self-report | 6-18 months | ***Correlations:***  Anxiety T1 🡪 Q x F T2: 0.07, p > .05  Anxiety T1 🡪 Q x F T3: 0.06, p > .05  Anxiety T1 🡪 Q x F T4: 0.14, p < .05  Anxiety T2 🡪 Q x F T3: 0.06, p > .05  Anxiety T2 🡪 Q x F T4:0.04, p > .05  Anxiety T3 🡪 Q x F T4: 0.07, p > .05  ***Parallel process latent growth model:***  No clear evidence that anxiety (13) is associated with Q x F of alcohol use. No numbers reported. | None for this analysis | 393 | N  N  N  N  N  N  Y | U |
| (25) | National Child Development Study, UK | 52 | Internalising behaviours (Health and Behaviour Checklists)  7, 11  Parent-report | Weekly quantity & harmful drinking (CAGE)  16, 23, 33  Self-report | 9-26 years | ***Hierarchical multiple regressions:***  (unstandardised)  Internalising (7) 🡪 quantity (16):  M: B (SE): -0.36 (0.13), p < .01  F: B (SE): -0.15 (0.08), p > .05  Internalising (11) 🡪 quantity (16):  M: B (SE): -0.64 (0.14), p < .001  F: B (SE): -0.08 (0.08), p > .05  Internalising (7) 🡪 quantity (23):  M: B (SE): -3.66 (0.76), p < .001  F: B (SE): -0.59 (0.26), p < .05  Internalising (11) 🡪 quantity (23):  M: B (SE): -2.54 (0.82), p < .01  F: B (SE): -0.01 (0.28), p > .05  Internalising (7) 🡪 quantity (33):  M: B (SE): -3.08 (0.67), p < .001  F: B (SE): -0.83 (0.28), p < .01  Internalising (11) 🡪 quantity (33):  M: B (SE): -2.77 (0.72), p < .001  F: B (SE): -0.76 (0.30), p < .01 | Social class, parent education years, parents read with child, social maladjustment, academic ability, externalising behaviour (age 7 for age 7 analyses and additionally at age 11 for age 11 analyses) | 4756-12772 | N  N  N  N  N  N  Y  Y  N  N  N  N | N  U |
| (26) | Healthy Schools and Drugs prevention program, Netherlands | 48 | Anxiety sensitivity (SURPS)  12-13, and 8, 20, 32 months later  Self-report | Alcohol use and binge drinking  12-13, and 8, 20, and 32 months later  Self-report | 8-32 months | ***Correlations:***  Anxiety sensitivity (T0) 🡪 Alcohol use (T1, T2, T3): −.10 (p < .05), −.07, −.08 (p > .05)  Anxiety sensitivity (T0) 🡪 Binge (T1, T2, T3):  −.09, −.08, −.09 (p < .05)  Anxiety sensitivity (T1) 🡪 Alcohol use (T2, T3):  −.10 (p < .05), −.07 (p > .05)  Anxiety sensitivity (T1) 🡪 Binge (T2, T3):  −.07, −.06 (p < .05)  Anxiety sensitivity (T2) 🡪 Alcohol use (T3):  −.07 (p > .05)  Anxiety sensitivity (T2) 🡪 Binge (T3):  −.08 (p > .05)  ***Cross-lagged models:*** (standardised beta)  Anxiety sensitivity (T0) 🡪 Alcohol use, binge (T1): 05, −.00, p >.05  Anxiety sensitivity (T1) 🡪 Alcohol use, binge (T2):  -.05, .01, p >.05  Anxiety sensitivity (T2) 🡪 Alcohol use, binge (T3): −.03, −.03, p >.05 | Sex and education level | 853-979 | N, N  N, N  Y, Y | U, U |
| (27) | Healthy Schools and Drugs prevention program, Netherlands | 48 | Anxiety sensitivity (SURPS)  12-13  Self-report | Lifetime prevalence of alcohol use  20 months later  Self-report | 20 months | ***Structural equation modelling. Cross-lagged paths:***  Anxiety 🡪 alcohol use  Standardised beta: -.012, p = .567 | Sex and education level | 648-758 | Y | E |
| (28) | Pittsburgh Youth Study, USA | 100 | Generalised anxiety and social anxiety (CBCL, YSR, TRF)  6  Parent-, teacher- and self-report | First alcohol problem (DIS)  20  Self-report | 14 years | ***Survival analysis:***  GAD 🡪 time from 1st use to 1st problem  OR [95% CI]: 1.03 [0.92 to 1.15] SAD 🡪 time from 1st use to 1st problem  OR [95% CI]: 1.03 [0.91 to 1.17] | Delinquency, interaction of anxiety and time, interaction of anxiety and delinquency | 503 | Y  Y | E  E |
| (29) | Camden Youth Development Study, USA | 50 | Social and generalised anxiety symptoms (SCARED)  11  Self-report | Frequency of drinking alcohol    Every 4 months for 16 months  Self-report | 16 months | ***Multilevel models:***  Social anxiety 🡪 alcohol use frequency  0.00 (parameter estimate), p > .05  Generalised anxiety 🡪 alcohol use frequency  0.00 (parameter estimate), p > .05 | Age, gender, and race | 134 | Y  Y | U  U |
| (30) | From secondary schools in the state of Victoria, Australia | No info | Anxiety/depression symptoms (CIS)  14-17 (6 waves every 6 months)  Self-report | Alcohol abuse or dependence (CIDI)  24  Self-report | 10 years | ***Logistic regression:***  Anxiety/depression 🡪 alcohol abuse or dependence  1-2 waves: OR [95% CI]: 1.3 [1.2 to 1.4], p < .001  >2 waves: OR [95% CI]: 1.9 [1.7 to 2.0], p < .001 | Adolescent alcohol use, tobacco use, sex, school location, country of birth, parental education, tobacco and alcohol use, marital status, | 1758 | N  Y | P |
| (31) | Northern Finland Birth Cohort 1986 Study, Finland | 49 | Internalising problems (Rutter Scales)  8  Parent-, teacher-, self-report | Often drunk  15-16  Self-report | 7 years | ***Logistic regression:***  Internalising symptoms 🡪 frequent drunkenness  OR [95% CI] males 0.7 [0.5 to 1.1], p > .05  OR [95% CI] females 0.8 [0.6 to 1.1], p > .05 | Place of residence, family pattern, social status, parental alcohol use, and parental psychiatric disorders | 6349 | Y  Y | WN  WN |
| (32) | The pathways to desistance project, juvenile offenders, USA | 100 | Worry, physiological anxiety (RCMAS)  14-19  Self-report | Typical quantity of drinking, frequency of binge drinking, dependence  6 months later  Self-report | 6 months | ***Zero-inflated poisson regression analysis:***  Physiological anxiety 🡪 quantity of drinking  B (unstandardised) = .10, SE = .04, p = .001  Physiological anxiety 🡪 frequency of binging  B (unstandardised) = .04, SE = .02, p = .05  Physiological anxiety 🡪 and alcohol dependence  B (unstandardised) = .20, SE = .06, p = .002  Worry 🡪 quantity  B (unstandardised) = -.09, SE = .03, p = .001  Worry 🡪 frequency of bingeing  B (unstandardised) = -.04; SE = .01, p = .002  Worry 🡪 alcohol dependence  B (unstandardised) = - .14, SE = .05, p = .002 | Race/ethnicity, Wave 1 alcohol use, self-reported offending, and PST (proportion of supervised time) | 818 | Y  Y  Y  Y  Y  Y | P  WP  P  N  N  N |
| (33) | Pittsburgh Youth Study, USA | 100 | Anxiety/withdrawal (YSR, TRF, CBCL)  13  Parent-report, teacher-report, self-report | Alcohol abuse and dependence (DIS)  20, 25  Self-report | 12 years | ***Zero-inflated poisson regression:***  Anxiety/withdrawal 🡪 alcohol use disorder  RRR [95% CI]: .858 [.774 to .952], p = .004  ***Multinomial Logistic Regression:***  Anxiety/withdrawal symptoms 🡪 dependence  RRR [95% CI] = .674 [.512 to .890], p = .005  Anxiety/withdrawal symptoms 🡪 alcohol abuse RRR [95% CI] = .814 [.610 to 1.085], p = .161 | Age, minority status, socioeconomic status, family history of  alcohol/drug problems, history of alcohol use and alcohol-related problems at the time of the psychopathology variables | 506 | Y  N | N |
| (34) | California Families Project, USA | 50 | Internalising symptoms (MASQ)  14, 16  Self-report | Frequency of alcohol use  14, 16  Self-report | 2 years | ***Cross-lagged latent variable regression models:***  Standardised estimates of structural coefficients  Internalising symptoms (anxiety) 🡪 frequency of alcohol us: .06, p > .05  Internalising symptoms (anxious arousal) 🡪 frequency of alcohol use: .05, p < .05 | Gender, generational status, delinquency | 620 | N  Y | P |
| (35) | From secondary special education schools, Netherlands | 88 | Anxiety sensitivity (SURPS)  13  Self-report | Alcohol use (quantity x frequency) and problems (trajectories)  2 year follow up (6-8 months between waves)  Self-report | 6 months- 2 years | ***Multinomial logistic regression:***  Anxiety sensitivity 🡪 onset group  OR [95% CI]: 0.83 [0.48 to 1.42], p > .01  Anxiety sensitivity 🡪 early onset persistent drinking group  OR [95% CI]: 0.42 [0.35 to 0.77], p < .001  Anxiety sensitivity 🡪 persistent drinking group  OR [95% CI]: 0.51 [0.30 to 0.87]), no p value | No information | 378 | N  Y  N | N |
| (36) | Jyväskylä Longitudinal Study of Personality and Social Development, Finland | 53 | Anxiety (1 item)  8, 14  Teacher-report | Heavy use, frequency of drinking, binge drinking, problem drinking (LSQ and interview questions)  20, 27, 42  Self-report | 12-34 years | ***Regression:*** (standardised betas)  Anxiety (age 8) 🡪 heavy drinking at 20  M: beta = 0.14, p > .05; F: beta = 0.04, p > .05  Anxiety (age 8) 🡪 problem drinking at 27  M: beta = -0.15, p > .05; F: beta = 0.09, p > .05  Anxiety (age 8) 🡪 problem drinking at 42  M: beta = 0.06, p > .05; F: beta = -0.02, p >.05  Anxiety (age 8) 🡪 drinking frequency at 27  M: beta = 0.06, p > .05; F: beta = -0.06, p > .05  Anxiety (age 8) 🡪 drinking frequency at 42  M: beta = -0.03, p > .05; F: beta = -0.08, p > .05  Anxiety (age 8) 🡪 binge drinking at 27  M: beta = 0.15, p > .05; F: beta = -0.07, p > .05  Anxiety (age 8) 🡪 binge drinking at 42  M: beta = -0.03, p > .05; F: beta = -0.06, p > .05  Anxiety (age 8) 🡪 CAGE score at 27  M: beta = 0.07, p > .05; F: beta = 0.09, p > .05  Anxiety (age 8) 🡪 CAGE score at 42  M: beta = 0.08, p > .05; F: beta = 0.00, p > .05  Anxiety (age 14) 🡪 heavy drinking (age 20)  M: beta = -0.24, p < .01; F: beta = -.07, p > .05  Anxiety (age 14) 🡪 problem drinking (27)  M: beta = .01, p > .05; F: beta = .00, p > .05  Anxiety (age 14) 🡪 problem drinking (42)  M: beta = -.14, p > .05; F: beta = -.11, p > .05  Anxiety (age 14) 🡪 frequent drinking (age 27)  M: beta = -.15, p >.05; F: beta = -0.20, p < .01  Anxiety (age 14) 🡪 frequent drinking (age 42)  M: beta = -.01, p > .05; F: beta = -0.19, p < .01.  Anxiety (age 14) 🡪 binge drinking (age 27)  M: beta = -.16, p > .05; F: beta = -.13, p > .05  Anxiety (age 14) 🡪 binge drinking (age 42)  M: beta = -.16, p > .05; F: beta = -.13, p > .05  Anxiety (age 14) 🡪 CAGE score at 27  M: beta = -.09, p > .05; F: beta = -.03, p > .05  Anxiety (age 14) 🡪 CAGE score at 42  M: beta = -.03, p > .05; F: beta = -.12, p > .05 | Socioeconomic status, child-centred parenting, parental drinking, smoking mother, social activity, constructiveness, compliance, aggression, low self-control, school success | 290-347 | N, N  N, N  N, N  N, N  N, N  N, N  N, N  N, N  N, N  Y, Y  Y, Y  N, N  N, N  N, N  Y, Y  N, N  N, N  N, N | U, U  U, U  U, U |
| (37) | Jyväskylä Longitudinal Study of Personality and Social Development, Finland | 53 | Anxiety (3 items)  8, 14  Peer nomination and teacher-report | Social drinking, problem drinking, controlled drinking (CAGE)  26  Self-report | 12-18 years | ***Product moment correlations:***  Social anxiety age 8 (peer)🡪 problem drinking  M: -.15, p < .05; F: .24, p < .01  Social anxiety age 8 (peer)🡪 social drinking  M: -.20, p < .05; F: -.13, p > .05  Social anxiety age 8 (peer)🡪 controlled drinking  M: -.18, p < .05; F: -.02, p > .05  Social anxiety age 8 (teacher) 🡪 problem drinking  M: .10, p > .05; F: .17, p < .05  Social anxiety age 8 (teacher) 🡪 social drinking  M: .00, p > .05; F: -.07, p > .05  Social anxiety age8 (teacher) 🡪 controlled  M: -.16, p < .05; F: -.10, p > .05  Social anxiety age 14 (peer)🡪 problem drinking  M: -.25, p < .001; F: .15, p < .05  Social anxiety age 14 (peer)🡪 social drinking  M: -.07, p > .05; F: -16, p < .05  Social anxiety age 14 (peer) 🡪 controlled drinking  M: -16, p < .05l F: -.01, p > .05  Social anxiety age 14 (teacher)🡪 problem drinking  M: -.05, p > .05; F: .16, p < .05  Social anxiety age 14 (teacher) 🡪 social drinking  M: -.22, p < .01; F: -.06, p > .05  Social anxiety age 14 (teacher) 🡪 controlled  M: -.15, p <.05; F: -.05, p > .05  ***Path analysis:***  Females: anxiety T1, problem drinking T3:  Beta = .22, p < .05  Males: anxiety T2, problem drinking T3:  Beta = -.21, p < .01 | No information | 242-311 | N  N  N  N  N  N  N  N  N  N  N  N  Y  Y | P  N |
| (38) | Finn Twin12 study, Finland | 51 | Social anxiety (MPNI)  12  Peer-, parent- and teacher-report, self-report | Drinking frequency, alcohol dependence (SSAGA)  14, 17, 22  Self-report | Trajectories (max 10 years) | ***Latent growth curve analysis:***  Peer rated social anxiety 🡪 drinking frequency  Slope: -.24, p < .05  Parent rated social anxiety 🡪 alcohol use  Slope: -.06, p > .05  Teacher rated social anxiety 🡪 alcohol use  Slope: -.09, p > .05  ***Regressions***: (unstandardized betas)  Social anxiety (peer) 🡪 alcohol dependence 14  -.004, p < .001  Social anxiety (peer) 🡪 alcohol dependence 22  -.01, p = .001  Social anxiety (parent) 🡪 alcohol dependence 14  -.02, p > .2  Social anxiety (parent) 🡪 alcohol dependence 22  -.02, p > .2  Social anxiety (teacher)🡪 alcohol dependence 14  -.02, p > .2  Social anxiety (teacher)🡪 alcohol dependence 22  -.07, p > .2 | No information | 1225-1906 | N  Y  N  N  N  N  Y  N  N | N  E |
| (39) | Community sample, USA | 45 | Internalising problems (YSR)  11-12  Self-report | Alcohol use (YSR of Achenbach Assessment)  12-13, 13-14  Self-report | 1-2 years | ***Structural equations model (SEM) with latent variable interactions:***  Estimated standardised path coefficients  Internalising problems 🡪 alcohol use a year later  -.21, p < .05  Internalising problems 🡪 alcohol use 2 years later  -.03, p > .05 | Age | 387 | N  Y | U |
| (40) | From a primary prevention study, USA | 39 | Anxiety sensitivity (ASI), trait anxiety (STPI)  16-24  Self-report | Alcohol use disorder (SCID-NP)  18-26  Self-report | 2 years | ***Hierarchical logistic regression:***  Total ASI score 🡪 AUD  B (unstandardised) = .09, SE = .03, p = .007  Physical subscale 🡪 AUD  B (unstandardised) = .15, SE = .06, p = .007  Cognitive subscale 🡪 AUD  B (unstandardised) = .29, SE = .14, p = .04  Social subscale 🡪 AUD  B (unstandardised) = .26, SE = .14, p = .05  Trait anxiety 🡪 AUD  B (unstandardised) = .06, SE = .07, p = .36 | Experimental condition, trait anxiety, gender, ASI × gender | 295 | Y  N  N  N  N | P |
| (41) | American Indian Research data, USA | No info | Internalising behaviours (CBCL)  11  Self-report, parent-report | Alcohol use disorder (SSAGA-II)  19-20  Self-report | 9 years | ***Logistic regression:***  Internalising behaviour 🡪 alcohol use disorders  OR [95% CI]: 0.96 [.91 to 1.02], p > .05 | Gender, income | 281 | Y | WN |
| (42) | Community sample, USA | 47 | Internalising behaviour problems (RBPC)  11-15  Maternal-report, teacher report | Alcohol use (MAST, NYS)  17-22  Self-report | 6 years | ***Hierarchical multiple regression:***  Internalising behaviour problems 🡪 alcohol use Beta weights = -0.042, p = ns | Gender, externalising problems | 185-187 | Y | U |
| (43) | Longitudinal community sample (1/2 parental alcoholism), USA | 52 | Internalising symptoms (CBCL)  12-16  Self-report, maternal-report | Quantity and frequency of alcohol use, problem alcohol use  13-17  Self-report | 1 year | ***Manifest variable structural equation models:*** (Standardised path coefficient)  Internalising 🡪 alcohol use (adolescent)  -.01, p > .05  Internalising 🡪 alcohol use (maternal)  0.06, p > .05 | Adolescent age, parental alcoholism | 216 | Y  N | U |
| (44) | Young-HUNT 1, and Young-HUNT 2, Norway | 46 | Anxiety/depression (SCL 90-R, SCL-5)  13-15  Self-report | Frequent alcohol use  17-19  Self-report | 4 years | ***Logistic regression:***  Anxiety/depression symptoms 🡪 alcohol use total [OR 95% CI]: 0.9 [0.7 to 1.0] | Age, attention problems, conduct problems, pain and tension problems, early alcohol intoxication | 2399 | Y | WN |
| (45) | Random sample from secondary schools, Australia | 50 | Anxiety/depression symptoms (CIS-R)  14/15–17 (2 waves every 6 months)  Self-report | Alcohol use disorder symptom classes (CIDI)  24  Self-report | 10 years max | ***Latent class analysis:***  Anxiety/depression 🡪 moderate (vs. mild) AUD  OR [95% CI]: 1.9 [1.2 to 3.1], p < .05  Anxiety/depression 🡪 severe (vs. mild) AUD  OR [95% CI]: 2.5 [1.3 to 5.0], p < .05  Anxiety/depression 🡪 severe (vs. moderate) AUD  OR [95% CI]: 0.75 [0.31 to 1.8], p > .05 | Age of alcohol initiation, alcohol use and problems, smoking, cannabis use, antisocial behaviour, school location, parental drinking, smoking, separation, education | 1203 | N  Y  N | P |
| (46) | Victoria Healthy Youth Survey, Canada | 49 | Internalising symptoms (BCFPI)  12/13, 14/15, 16/17  Self-report | Heavy episodic drinking, alcohol related harms (Harmful Effects of Alcohol Scale)  12/13, 14/15, 16/17, 18/19  Self-report | 2 years | ***Cross-lagged panel models:***  Internalising (12/13) 🡪 HED (14/15), p > .05  Internalising (14/15) 🡪 HED (16/17), p > .05  Internalising (16/17) 🡪 HED (18/19), p > .05  Standardised estimates:  Internalising (14/15) 🡪 alcohol harms (16/17)  .12, p < .001  Internalising (16/17) 🡪 alcohol harms (18/19)  .10, p < .001 | Mother’s education as a proxy for SES | 657-662 | N  N  Y  N  Y | U  P |
| (47) | The Northern Swedish Cohort Study, Sweden | 52 | Anxiousness (DSM-5)  16  Self-report | Drinking trajectories (frequency, consumption)  16, 18, 21, 30, 42  Self-report | Trajectories (26 years max) | ***Multinomial logistic regression (also with latent class growth analysis):***  Anxiousness 🡪 ordinary drinking  OR [95% CI]: 1.97 [1.08 to 3.60], p < .05  Anxiousness 🡪 early onset low  OR [95% CI]: 2.43 [1.21 to 4.88], p < .05  Anxiousness 🡪 early onset moderate  OR [95% CI]: 2.84 [1.56 to 5.15], p < .05  Anxiousness 🡪 early onset high  OR [95% CI]: 3.59 [1.89 to 6.82], p < .05  Anxiousness 🡪 late onset low trajectory  OR [95% CI]: 1.54 [0.72 to 3.32], p > .05 | Gender, social class of the parents | 1010 | N  N  N  Y  N | P |
| (48) | Black adolescents with asthma, USA | 34 | Anxiety symptoms (MASC-10)  11-19  Self-report | Alcohol use frequency (from Adolescent Risk Behavior Survey)  12-20  Self-report | 1 year | ***Logistic regression:***  Anxiety symptoms 🡪 alcohol use  OR [95% CI]: 1.12 [1.02 to 1.23], p < .05 | Alcohol use T1. Age, gender, negative coping, asthma symptoms, concern, severity were removed from final model as not significant | 110 | Y | P |
| (49) | Northwestern-UCLA Youth Emotion Project, USA | 31 | Anxiety disorders (SCID-I/NP)  16  Self-report | Alcohol use disorder (SCID-I/NP)  1-4 years later  Self-report | 1-4 years | ***Logistic regression:***  Anxiety disorders 🡪 alcohol use disorder onset  OR [95% CI]: 2.71 [1.39 to 5.29], p < .01  Social anxiety disorder 🡪 AUD  OR [95% CI]:2.52 [1.10 to 5.80], p < .05  Panic disorder 🡪 AUD p > .27  OCD 🡪 AUD p > .27  GAD 🡪 AUD p > .27 | Gender | 420-627 | Y  Y  Y  Y  Y | P  P  U  U  U |
| (50) | Christchurch Health and Development Study, New Zealand | 50 | Anxiety disorders (DISC supplemented by DSM-III-R)  15-16  Self-report | Alcohol abuse/dependence (CIDI)  Between 16 and 21, annually  Self-report | 1-6 years | ***Logistic regression:***  Anxiety disorders 🡪 alcohol dependence  p > .70 | Childhood sexual abuse, baseline alcohol abuse, deviant peer affiliations | 964 | Y | U |
| (51) | Early Developmental Stages of Psychopathology Study, Germany | No info | Anxiety disorders (DIA-X/M-CIDI)  14-24  Self-report | Regular use, hazardous use, abuse, dependence, alcohol use disorder (M-CIDI)  20 and 42 months later  Self-report | 4 years | ***Logistic regression:***  Panic disorder 🡪 at least regular use  OR [95% CI]: 0.6 [0.1 to 1.9] p > .05  Panic disorder 🡪 hazardous use  OR [95% CI]: 1.1 [0.3 to 3.6] p > .05  Panic disorder 🡪 abuse  OR [95% CI]: 2.4 [0.4 to 11.4] p > .05  Panic disorder 🡪 dependence  OR [95% CI]: 3.7 [0.8 to 15.9] p > .05  Panic disorder 🡪 any AUD  OR [95% CI]: 2.8 [0.8 to 9.1] p > .05  Panic attacks 🡪 regular use  OR [95% CI]: 1.8 [0.7 to 4.4], p > .05  Panic attacks 🡪 hazardous use  OR [95% CI]: 2.5 [1.1 to 5.8] p < .05  Panic attacks 🡪 alcohol abuse  OR [95% CI]: 2.7 [1.1 to 6.1], p < .05  Panic attacks 🡪 dependence  OR [95% CI]: 1.4 [0.2 to 8.7], p > .05  Panic attacks 🡪 any AUD  OR [95% CI]: 2.0 [0.8 to 4.7], p > .05  Social phobia 🡪 regular alcohol use  OR [95% CI]: 1.9 [1.0 to 3.4], p < .05  Social phobia 🡪 hazardous use  OR [95% CI]: 2.1 [1.2 to 3.8], p < .05  Social phobia 🡪 alcohol abuse  OR [95% CI]: 0.7 [0.3 to 1.3], p > .05  Social phobia 🡪 dependence  OR [95% CI]: 0.4 [0.1 to 1.4], p > .05  Social phobia 🡪 any AUD  OR [95% CI]: 0.6 [0.3 to 1.1], p > .05  GAD 🡪 regular use  OR [95% CI]: 1.5 [0.6 to 3.4], p > .05  GAD 🡪 hazardous use  OR [95% CI]: 1.4 [0.5 to 3.2], p > .05  GAD 🡪 abuse  OR [95% CI]: 0.7 [0 .2 to 2.3], p > .05  GAD 🡪 dependence  OR [95% CI]: 0.7 [0.1 to 3.5], p > .05  GAD 🡪 Any AUD  0.7 [0.2 to 2.0], p > .05  Specific phobia 🡪 regular use  OR [95% CI]: 0.8 [0.5 to 1.3], p > .05  Specific phobia 🡪 hazardous use  OR [95% CI]: 0.9 [0.5 to 1.4], p > .05  Specific phobia 🡪 abuse  OR [95% CI]: 1.1 [0.6 to 1.8], p > .05  Specific phobia 🡪 dependence  OR [95% CI]: 1.3 [0.6 to 2.4], p > .05  Specific phobia 🡪 Any AUD  OR [95% CI]: 1.1 [0.7 to 1.8], p > .05 | Age, gender, other mental disorders, substance use disorders and antisocial behaviour | 2548 | N  N  N  N  N  N  Y  N  Y  N  N  Y  N  Y  N  N  Y  N  Y  N  N  Y  N  Y  N | P  E  P  WN  E  E  E    E |

**Note:** **Count Result:** Y = Yes, N = No. **Evidence:** N = Negative, WN = Weak Negative, E = Equivocal, WP = Weak Positive, P = Positive, U = Unclassifiable.

**Anxiety Measures:** Diagnostic Interview Schedule for Children (DISC): 3; Munich‐Composite International Diagnostic Interview (M‐CIDI): 5; Kiddie Schedule for Affective Disorders and Schizophrenia (K-SADS): 4; Achenbach System of Empirically Based Assessment (ASEBA), Child Behaviour Checklist (CBCL)/Youth Self-Report (YSR)/Teacher’s Report Form (TRF)/Young Adult Self-Report (YASR): 13; Clinical Interview Schedule (CIS)/Clinical Interview Schedule-Revised (CIS-R): 3; Revised Child Anxiety and Depression Scale (RCADS): 1; Strengths and Difficulties Questionnaire (SDQ): 1; Longitudinal Interval Follow-up Evaluation (LIFE): 2; Structured Clinical Interview for DSM (SCID)/Structured Clinical Interview for DSM Non Patient (SCID-NP): 3; Social Phobia Inventory (SPIN): 1; Clinician rated diagnosis: 1; Diagnostic Interview Schedule III Revised (DIS-III-R): 2; Diagnostic Interview for Children and Adolescents-Revised (DICA-R): 1; Brief Symptom Inventory (BSI): 1; Health and Behaviour Checklists: 1; Substance Use Risk Profile Scale (SURPS): 3; Screen for Child Anxiety Related Disorders (SCARED): 1; Rutter Scales: 1; Revised Children's Manifest Anxiety Scale (RCMAS): 1; Mini-Mood and Anxiety Symptom Questionnaire (MASQ): 1; Multidimensional Peer Nomination Inventory (MPNI): 1; Anxiety Sensitivity Index (ASI): 1; Revised Behaviour Problem Checklist (RBPC): 1; Symptom Check List (SCL-5): 1; Brief Child and Family Phone Interview (BCFPI): 1; Anxiousness (based on the symptom clusters in DSM-5): 1; Multidimensional Anxiety Scale for Children (MASC- 10): 1; State-Trait Personality Inventory (STPI): 1; and 2 researcher constructed measures.

**Alcohol Measures:** Diagnostic Interview Schedule for Children (DISC): 2; Diagnostic Interview Schedule (DIS): 2; Munich‐Composite International Diagnostic Interview (M‐CIDI): 5; Longitudinal Interval Follow-up Evaluation (LIFE): 4; Structured Clinical Interview for DSM (SCID)/Structured Clinical Interview for DSM Non Patient (SCID-NP): 5; National Youth Survey (NYS): 3; Clinical Interview Schedule (CIS): 1; Youth Risk Behavior Surveillance System (YRBSS): 1; Adult Heath Survey: 1; Adolescent Health Survey: 1; Kiddie Schedule for Affective Disorders and Schizophrenia (K-SADS): 3; Measures adapted from Questionnaire for the Alcohol, Health, and Behavior study: 1; Drinking and Drug History Questionnaire: 1; Diagnostic Interview for Children and Adolescents-Revised (DICA-R): 1; Composite International Diagnostic Interview: 3; CAGE Questionnaire (cut-annoyed-guilty-eye): 1; Semi-Structured Assessment for the Genetics of Alcoholism (SSAGA): 2; Youth Self-Report (YSR): 1; Michigan Alcohol Screening Test (MAST): 1; Harmful Effects of Alcohol Scale: 1; Adolescent risk behaviour survey: 1; and 19 researcher constructed measures.

**References**

1. Abram KM, Zwecker NA, Welty LJ, Hershfield JA, Dulcan MK, Teplin LA. Comorbidity and continuity of psychiatric disorders in youth after detention: A prospective longitudinal study. Jama Psychiat. 2015;72(1):84-93.

2. Asselmann E, Wittchen HU, Lieb R, Hofler M, Beesdo-Baum K. Associations of fearful spells and panic attacks with incident anxiety, depressive, and substance use disorders: a 10-year prospective-longitudinal community study of adolescents and young adults. J Psychiatr Res. 2014;55:8-14.

3. Behrendt S, Beesdo-Baum K, Zimmermann P, Höfler M, Perkonigg A, Bühringer G, et al. The role of mental disorders in the risk and speed of transition to alcohol use disorders among community youth. Psychol Med. 2011;41(5):1073-85.

4. Bruckl TM, Wittchen HU, Hofler M, Pfister H, Schneider S, Lieb R. Childhood separation anxiety and the risk of subsequent psychopathology: Results from a community study. Psychother Psychosom. 2007;76(1):47-56.

5. Buckner JD, Schmidt NB, Lang AR, Small JW, Schlauch RC, Lewinsohn PM. Specificity of social anxiety disorder as a risk factor for alcohol and cannabis dependence. J Psychiatr Res. 2008;42(3):230-9.

6. Cerda M, Prins SJ, Galea S, Howe CJ, Pardini D. When psychopathology matters most: identifying sensitive periods when within-person changes in conduct, affective and anxiety problems are associated with male adolescent substance use. Addiction. 2016;111(5):924-35.

7. Cheng AT, Gau S-F, Chen TH, Chang J-C, Chang Y-T. A 4-Year longitudinal study on risk factors for alcoholism. Arch Gen Psychiat. 2004;61(2):184-91.

8. Colder CR, Scalco M, Trucco EM, Read JP, Lengua LJ, Wieczorek WF, et al. Prospective associations of internalizing and externalizing problems and their co-occurrence with early adolescent substance use. Journal of Abnormal Child Psychology. 2013;41(4):667-77.

9. Dahne J, Banducci AN, Kurdziel G, MacPherson L. Early adolescent symptoms of social phobia prospectively predict alcohol use. J Stud Alcohol Drugs. 2014;75(6):929-36.

10. Edwards AC, Latendresse SJ, Heron J, Cho SB, Hickman M, Lewis G, et al. Childhood internalizing symptoms are negatively associated with early adolescent alcohol use. Alcoholism: Clinical and Experimental Research. 2014;38(6):1680-8.

11. Englund MM, Egeland B, Oliva EM, Collins W. Childhood and adolescent predictors of heavy drinking and alcohol use disorders in early adulthood: A longitudinal developmental analysis. Addiction. 2008;103(Suppl1):23-35.

12. Englund MM, Siebenbruner J. Developmental pathways linking externalizing symptoms, internalizing symptoms, and academic competence to adolescent substance use. J Adolesc. 2012;35(5):1123-40.

13. Essau CA, Lewinsohn PM, Olaya B, Seeley JR. Anxiety disorders in adolescents and psychosocial outcomes at age 30. Journal of Affective Disorders. 2014;163:125-32.

14. Farmer RF, Gau JM, Seeley JR, Kosty DB, Sher KJ, Lewinsohn PM. Internalizing and externalizing disorders as predictors of alcohol use disorder onset during three developmental periods. Drug and Alcohol Dependence. 2016;164:38-46.

15. Frojd S, Ranta K, Kaltiala-Heino R, Marttunen M. Associations of social phobia and general anxiety with alcohol and drug use in a community sample of adolescents. Alcohol and alcoholism (Oxford, Oxfordshire). 2011;46(2):192-9.

16. Goodman A. Substance use and common child mental health problems: Examining longitudinal associations in a British sample. Addiction. 2010;105(8):1484-96.

17. Goodwin RD, Lieb R, Hoefler M, Pfister H, Bittner A, Beesdo K, et al. Panic attack as a risk factor for severe psychopathology. The American journal of psychiatry. 2004;161(12):2207-14.

18. Gorka SM, Shankman SA, Olino TM, Seeley JR, Kosty DB, Lewinsohn PM. Anxiety disorders and risk for alcohol use disorders: The moderating effect of parental support. Drug and Alcohol Dependence. 2014;140:191-7.

19. Haller M, Chassin L. The influence of PTSD symptoms on alcohol and drug problems: Internalizing and externalizing pathways. Psychological Trauma: Theory, Research, Practice, and Policy. 2013;5(5):484-93.

20. Hill KG, Hawkins JD, Bailey JA, Catalano RF, Abbott RD, Shapiro VB. Person-environment interaction in the prediction of alcohol abuse and alcohol dependence in adulthood. Drug Alcohol Depend. 2010;110(1-2):62-9.

21. Jester JM, Steinberg DB, Heitzeg MM, Zucker RA. Coping expectancies, not enhancement expectancies, mediate trauma experience effects on problem alcohol use: A prospective study from early childhood to adolescence. Journal of Studies on Alcohol and Drugs. 2015;76(5):781-9.

22. Jun HJ, Sacco P, Bright CL, Camlin EAS. Relations among internalizing and externalizing symptoms and drinking frequency during adolescence. Substance Use and Misuse. 2015;50(14):1814-25.

23. King SM, Iacono WG, McGue M. Childhood externalizing and internalizing psychopathology in the prediction of early substance use. Addiction. 2004;99(12):1548-59.

24. Mackie CJ, Castellanos-Ryan N, Conrod PJ. Personality moderates the longitudinal relationship between psychological symptoms and alcohol use in adolescents. Alcoholism: Clinical and Experimental Research. 2011;35(4):703-16.

25. Maggs JL, Patrick ME, Feinstein L. Childhood and adolescent predictors of alcohol use and problems in adolescence and adulthood in the National Child Development Study. Addiction. 2008;103:7-22.

26. Malmberg M, Kleinjan M, Overbeek G, Vermulst AA, Lammers J, Engels R. Are there reciprocal relationships between substance use risk personality profiles and alcohol or tobacco use in early adolescence? Addict Behav. 2013;38(12):2851-9.

27. Malmberg M, Kleinjan M, Vermulst AA, Overbeek G, Monshouwer K, Lammers J, et al. Do substance use risk personality dimensions predict the onset of substance use in early adolescence? A variable- and person-centered approach. Journal of Youth and Adolescence. 2012;41(11):1512-25.

28. Marmorstein NR, White HR, Loeber R, Stouthamer-Loeber M. Anxiety as a predictor of age at first use of substances and progression to substance use problems among boys. Journal of Abnormal Child Psychology. 2010;38(2):211-24.

29. Marmorstein NR. Interactions Between Internalizing Symptoms and Urgency in the Prediction of Alcohol Use and Expectancies Among Low-Income, Minority Early Adolescents. Substance abuse : research and treatment. 2015;9(Suppl 1):59-68.

30. McKenzie M, Jorm AF, Romaniuk H, Olsson CA, Patton GC. Association of adolescent symptoms of depression and anxiety with alcohol use disorders in young adulthood: findings from the Victorian Adolescent Health Cohort Study. Medical Journal of Australia. 2011;195(3):S27-S30.

31. Miettunen J, Murray G, Jones P, Maki P, Ebeling H, Taanila A, et al. Longitudinal associations between childhood and adulthood externalizing and internalizing psychopathology and adolescent substance use. Psychol Med. 2014;44(8):1727-38.

32. Nichter B, Chassin L. Separate dimensions of anxiety differentially predict alcohol use among male juvenile offenders. Addict Behav. 2015;50:144-8.

33. Pardini D, White HR, Stouthamer-Loeber M. Early adolescent psychopathology as a predictor of alcohol use disorders by young adulthood. Drug and Alcohol Dependence. 2007;88(SUPPL.1):S38-S49.

34. Parrish KH, Atherton OE, Quintana A, Conger RD, Robins RW. Reciprocal relations between internalizing symptoms and frequency of alcohol use: Findings from a longitudinal study of mexican-origin youth. Psychology of Addictive Behaviors. 2016;30(2):203-8.

35. Peeters M, Monshouwer K, van de Schoot R, Janssen T, Vollebergh WA, Wiers RW. Personality and the prediction of high-risk trajectories of alcohol use during adolescence. Journal of studies on alcohol and drugs. 2014;75(5):790-8.

36. Pitkanen T, Kokko K, Lyyra A-L, Pulkkinen L. A developmental approach to alcohol drinking behaviour in adulthood: A follow-up study from age 8 to age 42. Addiction. 2008;.103(Suppl1):pp.

37. Pulkkinen L, Pitkanen T. A prospective study of the precursors to problem drinking in young adulthood. Journal of Studies on Alcohol. 1994;55(5):578-87.

38. Savage JE, Kaprio J, Korhonen T, Pulkkinen L, Rose RJ, Verhulst B, et al. The effects of social anxiety on alcohol and cigarette use across adolescence: Results from a longitudinal twin study in Finland. Psychology of Addictive Behaviors. 2016;30(4):462-74.

39. Scalco MD, Colder CR, Hawk LW, Read JP, Wieczorek WF, Lengua LJ. Internalizing and externalizing problem behavior and early adolescent substance use: A test of a latent variable interaction and conditional indirect effects. Psychology of Addictive Behaviors. 2014;28(3):828-40.

40. Schmidt NB, Buckner JD, Keough ME. Anxiety sensitivity as a prospective predictor of alcohol use disorders. Behavior Modification. 2007;31(2):202-19.

41. Stanley LR, Miller KA, Beauvais F, Walker PS, Walker R. Predicting an alcohol use disorder in urban American Indian youths. Journal of Child & Adolescent Substance Abuse. 2014;23(2):101-8.

42. Steele RG, Forehand R, Armistead L, Brody G. Predicting alcohol and drug use in early adulthood: The role of internalizing and externalizing behavior problems in early adolescence. American Journal of Orthopsychiatry. 1995;.65(3):pp.

43. Stice E, Barrera M, Jr., Chassin L. Prospective differential prediction of adolescent alcohol use and problem use: Examining the mechanisms of effect. Journal of Abnormal Psychology. 1998;107(4):616-28.

44. Strandheim A, Bratberg GH, Holmen TL, Coombes L, Bentzen N. The influence of behavioural and health problems on alcohol and drug use in late adolescence - a follow up study of 2 399 young Norwegians. Child and Adolescent Psychiatry and Mental Health. 2011;5.

45. Swift W, Slade T, Carragher N, Coffey C, Degenhardt L, Hall W, et al. Adolescent predictors of a typology of DSM-5 alcohol use disorder symptoms in young adults derived by latent class analysis using data from an Australian cohort study. Journal of Studies on Alcohol and Drugs. 2016;77(5):757-65.

46. Thompson KD, Leadbeater BJ, Ames ME. Reciprocal effects of internalizing and oppositional defiance symptoms on heavy drinking and alcohol-related harms in young adulthood. Substance Abuse: Research and Treatment. 2015;9:21-31.

47. Virtanen P, Nummi T, Lintonen T, Westerlund H, Hagglof B, Hammarstrom A. Mental health in adolescence as determinant of alcohol consumption trajectories in the Northern Swedish Cohort. International Journal of Public Health. 2015;63(3):335-42.

48. Weekes JC, Cotton S, McGrady ME. Predictors of substance use among black urban adolescents with asthma: A longitudinal assessment. Journal of the National Medical Association. 2011;103(5):392-8.

49. Wolitzky-Taylor K, Bobova L, Zinbarg RE, Mineka S, Craske MG. Longitudinal investigation of the impact of anxiety and mood disorders in adolescence on subsequent substance use disorder onset and vice versa. Addict Behav. 2012;37(8):982-5.

50. Woodward LJ, Fergusson DM. Life course outcomes of young people with anxiety disorders in adolescence. Journal of the American Academy of Child & Adolescent Psychiatry. 2001;40(9):1086-93.

51. Zimmermann P, Wittchen HU, Höfler M, Pfister H, Kessler RC, Lieb R. Primary anxiety disorders and the development of subsequent alcohol use disorders: A 4-year community study of adolescents and young adults. Psychol Med. 2003;33(7):1211-22.
